# UBXN9 governs GLUT4-mediated spatial confinement of RIG-I-like receptors and signaling

**DOI:** 10.21203/rs.3.rs-3373803/v1

**Published:** 2024-06-04

**Authors:** Penghua Wang, Andrew Harrison, Duomeng Yang, Jason Cahoon, Tingting Geng, Ziming Cao, Timofey Karginov, Conner Chiari, Xin Li, Yibing Qyang, Anthony Vella, Zhichao Fan, Sivapriya Kailasan Vanaja, Vijay Rathinam, Carol Witczak, Jonathan Bogan

**Affiliations:** University of Connecticut Health Center; University of Connecticut Health Center; UConn Health; University of Connecticut Health Center; UConn Health; University of Connecticut Health Center; UConn Health; University of Connecticut Health Center; Yale School of Medicine; Yale School of Medicine; School of Medicine, University of Connecticut; UConn Health; UConn Health School of Medicine; UConn Health School of Medicine; Indiana University School of Medicine; Yale School of Medicine

**Keywords:** GLUT4, UBXN9, RIG-I like receptor, Glucose, Metabolism

## Abstract

The cytoplasmic RIG-I-like receptors (RLRs) recognize viral RNA and initiate innate antiviral immunity. RLR signaling also triggers glycolytic reprogramming through glucose transporters (GLUTs), whose role in antiviral immunity is elusive. Here, we unveil that insulin-responsive GLUT4 inhibits RLR signaling independently of glucose uptake in adipose and muscle tissues. At steady state, GLUT4 is docked at the Golgi matrix by ubiquitin regulatory X domain 9 (UBXN9, TUG). Following RNA virus infection, GLUT4 is released and translocated to the cell surface where it spatially segregates a significant pool of cytosolic RLRs, preventing them from activating IFN-β responses. UBXN9 deletion prompts constitutive GLUT4 trafficking, sequestration of RLRs, and attenuation of antiviral immunity, whereas GLUT4 deletion heightens RLR signaling. Notably, reduced GLUT4 expression is uniquely associated with human inflammatory myopathies characterized by hyperactive interferon responses. Overall, our results demonstrate a noncanonical UBXN9-GLUT4 axis that controls antiviral immunity via plasma membrane tethering of cytosolic RLRs.

## INTRODUCTION

The retinoic acid inducible gene I (RIG-I)-like receptors (RLRs), comprised of RIG-I, melanoma differentiation-associated protein 5 (MDA5) and Laboratory of Genetics and Physiology 2 (LGP2), are a family of cytosolic pattern recognition receptors (PRRs) that sense viral RNA and initiate an early antiviral immune response to numerous RNA viruses. Following the sensing of distinct viral RNA structures, RIG-I and MDA5 utilize the conserved mitochondrial antiviral-signaling protein (MAVS) to initiate a signaling cascade leading to the activation of transcription factors IRF3 and NF-κB and subsequent transcription of type I/III interferon (IFN-I/III) and proinflammatory cytokine genes ^1^.

However, abnormal activation of RLRs is associated with autoimmune diseases and interferonopathies such as Type-I diabetes ^2, 3^, Aicardi–Goutières syndrome and dermatomyositis ^4, 5^, emphasizing that RLRs must be strictly regulated for discrimination between pathogenic RNA and unnecessary sterile responses. To date, post-translational modifications (PTMs) of the receptors themselves or the downstream signaling proteins are the predominant mechanisms by which RLRs are controlled. Whether additional regulatory strategies exist upstream of RLR activation has not been thoroughly explored.

In addition to cytokine production, activation of PRRs leads to metabolic reprogramming characterized by enhanced glucose influx, glycolysis, disruption of the tricarboxylic acid (TCA) cycle and accumulation of metabolites ^6^. Similarly, RNA viruses (sensed by RLRs) also promote glucose uptake ^7, 8^, where metabolites feedback from glycolysis ^9^, the pentose phosphate pathway ^10^, the hexosamine biosynthesis pathway ^11^ and the TCA cycle ^12^ to intersect at MAVS to suppress RLR signaling. This rewiring of cellular metabolism hinges on glucose transporters (GLUTs), which belong to the solute carrier (SLC) family of nutrient transporters ^13^. The relationship between GLUTs and immune function have been best described for GLUT1, which facilitates extensive glycolytic reprogramming in innate immune cells following Toll-like receptor (TLR) activation ^14, 15^. However, despite their importance to prompting glucose metabolism, the role of other GLUTs in innate immune responses is entirely unknown. Further, whether metabolites or glycolytic proteins regulate RLRs directly remains an intensive area of study.

GLUT4 is the predominant, insulin/muscle contraction-regulated glucose transporter in adipose, skeletal, and cardiac muscle tissues, and is essential for the maintenance of whole-body glucose homeostasis ^16^. In the basal state, GLUT4 is primarily sequestered within intracellular GLUT4 storage vesicles (GSVs), which are tethered to the Golgi matrix by a direct interaction with the Golgi-associated ubiquitin regulatory X (UBX) domain-containing protein UBXN9 (also known as TUG) ^17,18^. Insulin triggers site-specific endoproteolytic cleavage of UBXN9 to release GSVs for transport to the plasma membrane; thus, deletion of UBXN9 increases constitutive trafficking and surface residence of GLUT4, resembling the acute insulin effect on GLUT4 trafficking ^19^. Therefore, UBXN9-mediated GLUT4 tethering is the dominant mechanism for ensuring appropriate insulin signaling. Intriguingly, whether GLUT4 has any other biological function is entirely unknown.

Here, we uncover a new role of the UBXN9-GLUT4 axis in the regulation of RLR signaling, independent of GLUT4’s canonical function as a glucose transporter. We find that, in contrast to the current dogma stating PRR effector responses are supported by GLUTs, GLUT4 specifically sequesters RLRs to the plasma membrane, thereby spatially segregating them from downstream signaling components and curtailing the primary IFN response. On the other hand, UBXN9 tethers GLUT4 to the Golgi matrix, thus maintaining normal RLR signaling. This study reveals GLUT4 as a negative regulator of RLR signaling and establishes the plasma membrane-mediated partitioning of RLRs as a strategy to ensure signaling fidelity of cytoplasmic RNA sensors. Critically, this novel mechanism could operate in autoimmune diseases, where GLUT4 is reduced in patients with autoinflammatory myopathies, coinciding with overexpression of RLRs and interferon stimulated genes (ISGs). Hence, new therapies for autoinflammatory myopathies targeting the UBXN9-GLUT4-RLR axis could prevent damaging inflammation.

## RESULTS

### UBXN9 promotes RLR signaling and inhibits RNA virus infection

To understand the function of the UBXN9-GLUT4 axis in regulating RLR signaling, we utilized the mouse myoblast cell line, C2C12, which recapitulates many aspects of primary mouse myotube biology ^20^.

Moreover, skeletal muscle myocytes express a high level of GLUT4 ^21^ and sense RNA viruses through RLRs ^22, 23^. We genetically deleted UBXN9 (*Ubxn*9^−/−^; KO) using CRISPR-Cas9 technology (Supplemental Fig. 1a) and compared the RLR responses in *Ubxn*9^−/−^ to wild-type control cells (*Ubxn9*^+/+^; WT) generated with a non-targeting gRNA. Knockout of *Ubxn9* led to reduced *Ifnb1* mRNA and IFN-β protein levels throughout the course of RIG-I activation by 5’ triphosphate hairpin RNA (3p-hpRNA) as compared to its WT counterpart (Supplemental Fig. 1b, c). However, IFN-β expression was comparable between *Ubxn9*^+/+^ and *Ubxn9*^−/−^ cells treated with interferon-stimulatory DNA (ISD), which activates cyclic GMP-AMP (cGAMP) synthase-stimulator of interferon genes (cGAS-STING) signaling (Supplemental Fig. 1d). These data demonstrated that UBXN9 regulates RIG-I, but not cGAS signaling.

**Figure 1:**
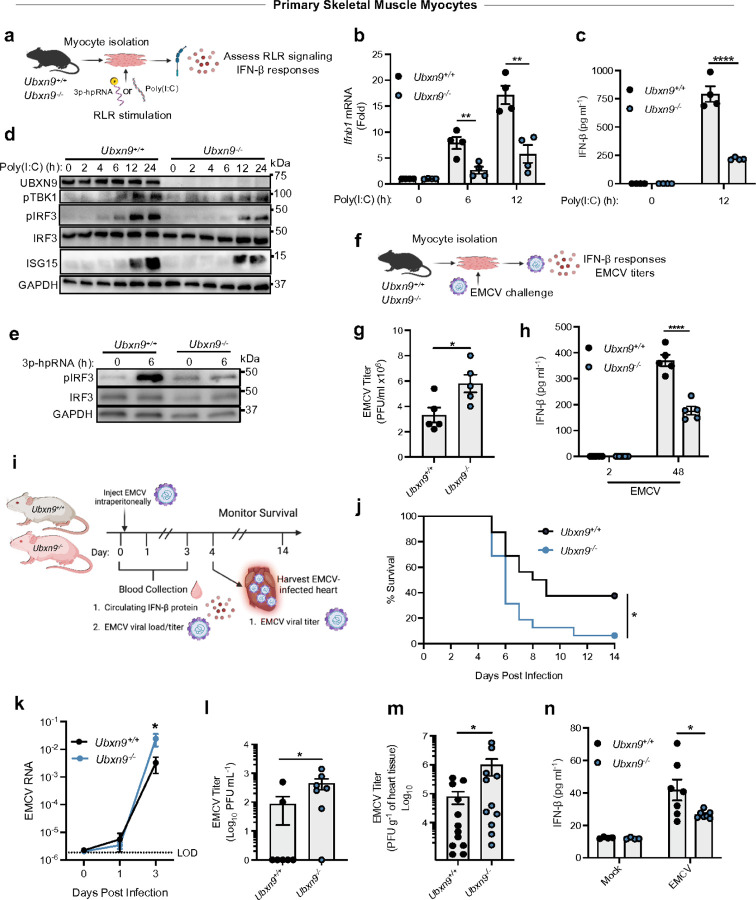
UBXN9 positively regulates RLR signaling in myocytes. **a,** Outline of protocol for assessing RIG-I-like receptor (RLR) responses in primary myocytes from *Ubxn9*^+/+^ and *Ubxn9*^−/−^ mice. **b, c,** cellular *Ifnb1* mRNA levels in (**b**) and IFN-β protein levels secreted (**c**) from mouse primary myocytes (n=4 mice/group) transfected with poly(I:C). **d, e,** immunoblots of primary interferon response proteins (**d**) and phosphorylated IRF3 (p-IRF3) (**e**) in mouse primary myocytes treated with poly(I:C) or 3p-hpRNA, respectfully. **f**, outline of protocol for assessing antiviral responses in primary myocytes isolated from (**a**). **g, h**, viral titers (**g**) and IFN-β protein concentrations (**h**) in the culture supernatant of primary myocytes (n=5 mice/group) infected with EMCV for 48h (MOI: 0.1). (PFU: plaque forming unit). **i**, *in vivo* timeline and protocol for assessing antiviral responses in *Ubxn9*^+/+^ and *Ubxn9*^−/−^ mice. **j**, Kaplan-Meier survival curve of age- and sex-matched mice (n=16 mice/group) from (**i**) infected with EMCV (100 PFU/mouse). **k-m**, viral RNA loads in the blood (n=4–8 mice/group) (k), viremia (n=7 mice/group) (**l**), and viral titers in the heart (n=11–12 mice/group) (**m**) from *Ubxn9*^+/+^ and *Ubxn9*^−/−^ mice infected with EMCV. Limit of detection (LOD) was established with uninfected animal blood. **n**, serum IFN-β levels 24h after EMCV infection (n=7 mice/group) (EMCV: 1,000 PFU/mouse). The results are representative of 2–3 independent experiments. Bar: mean ± SEM, *p<0.05, **p<0.01, ****p<0.0001 by two-way ANOVA (**b, c, h, k, n**), Log-Rank test (**j**) or unpaired Student’s *t-*test (all others).

To assess the immune function of UBXN9 in primary muscle myocytes and *in vivo*, we generated a tamoxifen-inducible global knockout mouse model by crossing *Ubxn9*^*flox/flox*^ (flanking Exon 5) with a Cre-recombinase-estrogen receptor T2 line (Cre^ERT2^) (Supplemental Fig. 2a). Adult Cre^ERT2^ -*Ubxn9*^flox/flox^ mice treated with tamoxifen (*Ubxn9*^−/−^) had nearly abolished UBXN9 protein in most tissues compared to controls treated with vehicle (corn oil) (*Ubxn9*^+/+^), except in the brain due to poor penetration of tamoxifen across the blood brain barrier (Supplemental Fig. 2b). Primary skeletal muscle myocytes from both mice expressed comparable levels of the signature transcription factors *Myf5* and *MyoG* for myoblast and myotube, respectively, indicating that UBXN9 is dispensable for myogenesis (Supplemental Fig. 2c-f). Conversely, *Ubxn9*^−/−^ myocytes showed decreased *Ifnb1* mRNA and IFN-β protein levels following MDA5 stimulation with polyinosinic-polycytidylic acid [Poly(I:C)] when compared to WT myocytes (Fig. 1a-c). In agreement with lower IFN-β production, *Ubxn9*^−/−^ myocytes had reduced phosphorylation of IRF3 (p-IRF3), the main transcription factor for *Ifnb1* (Fig. 1d-e), as well as impaired expression of interferon stimulated gene 15 (ISG15) at 24 hours post Poly(I:C) treatment, relative to *Ubxn9*^+/+^ controls (Fig. 1d).

Next, we tested whether the RLR-promoting function of UBXN9 is conserved in GLUT4-negative cells, such as macrophages. Bone marrow-derived macrophages (BMDMs) isolated from *Ubxn9*^+/+^ and *Ubxn9*^−/−^ mice (Supplemental Fig. 3a) revealed comparable *Ifnb1* mRNA inductions following stimulation with RLR agonists (Supplemental Fig. 3c). Moreover, UBXN9 deletion from a human lung epithelial cell line, A549, did not affect the IFN-β response to Poly(I:C) stimulation (Supplemental Fig. 3b, d). Thus, UBXN9 distinctively regulates RLR signaling in skeletal muscle—that canonically expresses GLUT4—and not in GLUT4^−^ macrophages or lung cells.

To address whether UBXN9 protects against infection with muscle tropic RNA viruses sensed by RLRs, we utilized encephalomyocarditis virus (EMCV, which specifically stimulates MDA5) and arthritogenic O'nyong nyong virus (ONNV, which activates both RIG-I and MDA5) ^22, 23, 24^. Consistent with compromised RLR signaling, UBXN9-deficient myocytes had higher EMCV titers and produced less IFN-β protein than their WT counterparts (Fig. 1f-h). We observed similar results with ONNV infection (Supplemental Fig. 1e, f). To validate the above-described *in vitro* studies, we utilized a mouse model of EMCV infection since EMCV has a tropism for the heart—which contains GLUT4^+^ cardiomyocytes—and is sensitive to type I IFNs ^23^ (Fig. 1i). Our recent work has demonstrated EMCV replication was ~ 100-fold higher in the heart than that in other tissues on day 4 post infection (p.i.) ^25^. Critically, *Ubxn9*^−/−^ mice were more susceptible to EMCV-induced lethality, accompanied by higher viral loads in the blood and heart relative to *Ubxn9*^+/+^ mice (Fig. 1j-m). Accordingly, knockout mice exhibited lower serum IFN-β concentrations (Fig. 1n). In an ONNV infection model, viral load in the skeletal muscle was relatively higher in *Ubxn9*^−/−^ mice compared to WT mice (Supplemental Fig. 1g, h). Collectively, these results argue that UBXN9 positively regulates RLR sensing of diverse RNA viruses *in vitro* and in mice.

### GLUT4 suppresses RLR signaling and promotes RNA virus infection

**Figure 2:**
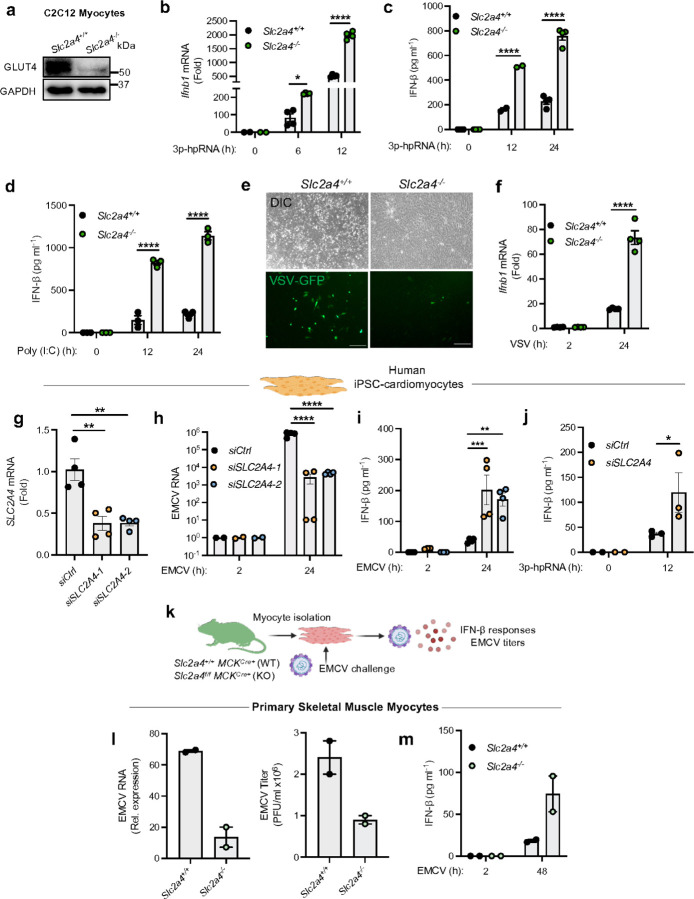
The glucose transporter GLUT4 suppresses RLR signaling. **a**, Immunoblot of GLUT4 in *Slc2a4*^+/+^ and *Slc2a4*^−/−^ C2C12 cells. **b, c,** cellular *Ifnb1* transcripts in (**b**) and secreted IFN-β protein concentrations (**c**) from *Slc2a4*^+/+^ and *Slc2a4*^−/−^ C2C12 cells (n=2–4 biological replicates) stimulated with 3p-hpRNA. **d**, IFN-β protein concentrations from *Slc2a4*^+/+^ and *Slc2a4*^−/−^ C2C12 cells (n=3 biological replicates) transfected with poly(I:C). **e,** fluorescence microscopic images of GFP in *Slc2a4*^+/+^ and *Slc2a4*^−/−^ C2C12 cells (n=4 biological replicates) infected with VSV-GFP (MOI:1) for 24 hr. DIC, differential interference contrast. Scale bar, 10µm. **f,** cellular *Ifnb1* mRNA levels from C2C12 cells infected with VSV-GFP as in (**e**). **g,** cellular *SLC2A4 (GLUT4)* transcripts from iPSCs-CMs transfected with either scrambled siRNA (*siCtrl*) or siRNA specific for GLUT4 (*siSLC2A4–1* or *siSLC2A4–2*). **h-i,** EMCV RNA levels in (**h**) and secreted IFN-β protein concentrations (**i**) from iPSC-CMs (n=4 biological replicates) siRNA-treated as in (**g**) and then infected with EMCV for 2 and 24h. EMCV (MOI: 0.05). **j,** secreted IFN-β protein concentrations from iPSC-CMs (N=3 independent batches of cells with 2–3 biological replicates per timepoint per batch) transfected with specific siRNA’s as in (**g**) and then stimulated with 3p-hpRNA. **k,** outline of protocol for assessing RLR responses in primary skeletal myocytes from *Slc2a4*^+/+^ *MCK*^*Cre+*^ (WT) and *Slc2a4*^*f/f*^ *MCK*^*Cre+*^ (GLUT4 KO) mice. **l,** EMCV viral load in and titers from primary myocytes (n=2 mice/group) isolated as in (**k**) infected for 48h (MOI: 0.1). **m,** IFN-β protein concentrations from primary myocytes infected with EMCV as in (**l**). The results are representative of 2–4 independent experiments (**a-j**) or one experiment (**k-m**). Bar: mean ± SEM. *p<0.05, **p<0.01, ***p<0.001, ****p<0.0001 by two-way ANOVA (**b-d, f, h-j,**) or unpaired Student’s *t-*test (**g**).

Since UBXN9 directly sequesters GLUT4 intracellularly and the IFN-β response was not influenced by UBXN9 in cells lacking GLUT4 (Supplemental Fig. 3), we postulated that GLUT4 also influences RLR signaling. Deletion of GLUT4 (gene symbol: *Slc2a4*) in C2C12 cells using CRISPR/Cas9 (Fig. 2a) significantly increased *Ifnb1* mRNA and IFN-β protein levels throughout the course of RIG-I/MDA-5 activation as compared to WT myocytes (*Slc2a4*^+/+^) (Fig. 2b-d). With a green fluorescence protein (GFP) reporter VSV strain, we observed less GFP and VSV-G protein expression in *Slc2a4*^−/−^ than those in *Slc2a4*^+/+^ cells, consistent with the significantly higher *Ifnb1* mRNA expression when GLUT4 was deleted (Fig. 2e, f, Supplemental Fig. 4a, b). We further recapitulated the same phenotypes with EMCV (Supplemental Fig. 4c, d). Accordingly, the increased IFN-β response was accompanied by higher IRF3 phosphorylation in *Slc2a4*^−/−^ cells infected with ONNV or EMCV as compared to its WT counterpart (Supplemental Fig. 4e, f). Thus, GLUT4 diverges from the dogma that glucose transporters promote PRR signaling ^26^.

In addition to skeletal muscle, GLUT4 is also the dominant GLUT expressed in cardiac tissue ^27^. To investigate the conservation of GLUT4-mediated RLR suppression in cardiac muscle cells, we differentiated cardiomyocytes from human induced pluripotent stem cells (hiPSCs-CMs) based on the modulation of Wnt signaling ^28^, silenced GLUT4 using siRNA and treated them with 3p-hpRNA or EMCV to activate RLRs (Supplemental Fig. 5a). hiPSC-CMs expressed the cardiac troponin T differentiation marker and GLUT4 mRNA was significantly reduced as compared to nontargeting control siRNA (Fig. 2g, Supplemental Fig. 5b). Notably, the knockdown of GLUT4 significantly decreased EMCV replication and augmented the IFN-β response when compared to control-treated hiPSC-CMs (Fig. 2h, i). Further, GLUT4 also suppressed RIG-I signaling as the stimulation of *siSLC2A4* hiPSC-CMs with 3p-hpRNA led to an elevated IFN-β response compared to *siCtrl* cells (Fig. 2j). Finally, we tested whether RLR responses are blunted by GLUT4 in primary myocytes derived from *Slc2a4*^*fl/fl*^ *MCK*^*Cre+*^ mice where GLUT4 expression is ablated by muscle creatine kinase (MCK) synthesis in skeletal and cardiac muscle ^29, 30^ (Fig. 2k). This permitted the direct assessment of GLUT4’s putative proviral role in muscle cells without the systemic metabolic disturbances accompanied by GLUT4 deletion *in vivo* ^30, 31^. Consistent with C2C12 cells and iPSC-CMs, GLUT4 deletion reduced EMCV replication and heightened IFN-β responses at 48 hours post-infection (Fig. 2l, m). These data suggest that GLUT4 suppression of RLRs is a highly conserved mechanism in mouse and human muscle cells.

### GLUT4-mediated RLR suppression is uncoupled from glucose influx and lactate accumulation

**Figure 3:**
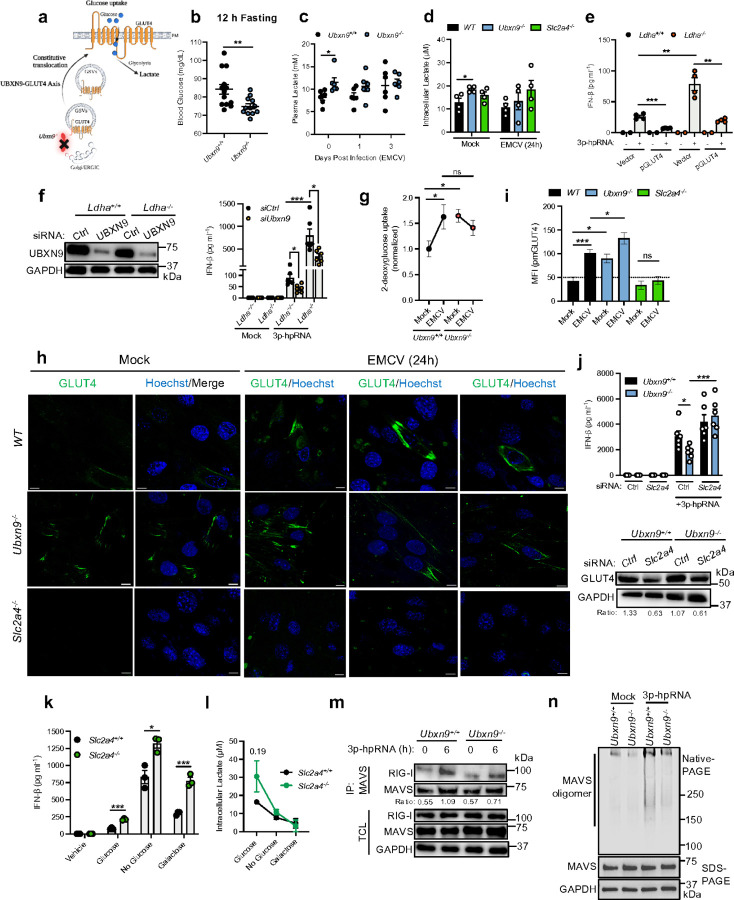
GLUT4-mediated RLR suppression is uncoupled from glucose influx and lactate accumulation. **a,** diagram illustrating constitutive GLUT4 trafficking to the plasma membrane in *Ubxn9*^−/−^ cells. **b,** blood glucose levels in age- and sex-matched *Ubxn9*^+/+^ and *Ubxn9*^−/−^ littermates (n=12–13 mice/group) after overnight fasting. **c,** plasma lactate concentrations in mice (n=6 mice/group) before and after infection with EMCV (100 PFU/mouse). **d,** intracellular lactate levels in C2C12 cells (n=4 biological replicates/group) before and after EMCV (MOI: 0.5). Mock, medium alone without virus. **e,** IFN-β protein concentrations from *Ldha*^+/+^ and *Ldha*^−/−^ C2C12 cells (n=4 biological replicates/group) that were transfected with an empty vector (Vector) or GLUT4 expression plasmid (pGLUT4) for 24 hr before 3p-hpRNA treatment for 12 hr. **f,** immunoblots of UBXN9 and GAPDH in *Ldha*^+/+^ and *Ldha*^−/−^ C2C12 myocytes transfected with Ctrl or *Ubxn9* siRNA for 48 hr (left) and IFN-β protein concentrations (right) following 3p-hpRNA transfection for 12 h. **g,** 2-deoxyglucose uptake (N=8–9 biological replicates/group) in *Ubxn9*^+/+^ and *Ubxn9*^−/−^ cells before and after EMCV infection (MOI: 0.5) for 24 hr. **h,** confocal microscopic images of exofacial GLUT4 and nuclear DNA (Hoechst) from C2C12 myocytes infected without (mock) or with EMCV (MOI: 0.5) for 24 hr. Scale bar, 10 μm). **i,** mean fluorescence intensity (MFI) (N=5–15 fields/group) of plasma membrane GLUT4 (pmGLUT4) from cells infected in (**h**). Mock, medium alone without virus. **j,** IFN-β protein (above) and immunoblot (below) from *Ubxn9*^+/+^ and *Ubxn9*^−/−^ cells (n=6 biological replicates/group) transfected with Ctrl or *Slc2a4* siRNAs for 48 hr, and then stimulated with 3p-hpRNA for 12 hr. Ratios denote GLUT4/GAPDH band densities for each sample. (n=6 biological replicates/group). Immunoblot samples were taken 48 hr after siRNA transfection. **k-l,** IFN-β protein (**k**) and intracellular lactate concentrations (**l**) from *Slc2a4*^+/+^ and *Slc2a4*^−/−^ C2C12 cells (n=3 biological replicates/group) grown in various sugar conditions and stimulated with 3p-hpRNA for 12 hr. Vehicle, transfection reagent only. **m,** endogenous MAVS co-immunoprecipitation (IP) from *Ubxn9*^+/+^ and *Ubxn9*^−/−^ C2C12 cells stimulated with 3p-hpRNA for 6 hr. Ratios denote RIG-I/MAVS band densities in IP sample. TCL, total cell lysate. **n,** MAVS filament formation in *Ubxn9*^+/+^ and *Ubxn9*^−/−^ cells stimulated with 3p-hpRNA for 6 hr. The results are representative of 2–4 independent experiments. Bar: mean ± SEM. *p<0.05, **p<0.01, ***p<0.001 by one-way ANOVA for (**g, i**), two-way ANOVA (**d**) or unpaired Student’s *t-*test for all others.

Until recently, glycolysis and RLR signaling pathways were thought to occur independently. It has now been reported that lactate—one of the chief by-products of glycolysis—is an inhibitor of RLR signaling by disrupting MAVS aggregation ^9,^, and MAVS itself coordinates glucose flux through the pentose-phosphate pathway (PPP) and the hexosamine biosynthesis pathway (HBP) ^10^. Based on the premise that UBXN9 tethers GLUT4 intracellularly, we reasoned that upon UBXN9 deficiency, GLUT4 would constitutively transport glucose resulting in increased intracellular/circulating levels of lactate, which then inhibits RLR signaling (Fig. 3a). In support of this possibility, deletion of lactate dehydrogenase A (*Ldha*^−/−^)—the end-stage glycolytic enzyme that catalyzes the conversion of pyruvate to lactate—increased IFN-β expression and reduced lactate release (Supplemental Fig. 6a-d). Moreover, global *Ubxn9*^−/−^ mice exhibited reduced glucose concentrations during fasting and after intraperitoneal injection of glucose, compared to *Ubxn9*^+/+^ counterparts, consistent with the muscle-specific *Ubxn9* deletion mouse model ^19^ (Fig. 3b, Supplemental Fig. 6e, f).

To first address this hypothesis, *Ubxn9*^+/+^ and *Ubxn9*^−/−^ mice were fasted before EMCV infection, and lactate levels monitored throughout the course of acute disease. Despite higher lactate levels in *Ubxn9*^−/−^ mice before infection, these levels equilibrated between WT and KO animals by 3 days post infection (Fig. 3c). Comparable trends in lactate were also noted in skeletal muscle cells (Fig. 3d). To rule out a direct role for lactate in our system, we overexpressed GLUT4 in *Ldha*^+/+^ and *Ldha*^−/−^ cells and treated them with 3p-hpRNA. Albeit significantly lower lactate, GLUT4 suppressed RLR responses equally in *Ldha*^−/−^ cells as those in WT control cells (Fig. 3e). Aligning with these results, silencing UBXN9, which effectively redistributes GLUT4 to the plasma membrane, markedly diminished IFN-β release from *Ldha*^+/+^ and *Ldha*^−/−^ cells, compared with *siCtrl* cells (Fig. 3f). While we consistently observed LDHA indirectly contributes (e.g., catabolism of pyruvate) to suppression of IFN-β responses, our knockdown data argues that GLUT4 is upstream of lactate inhibition in the hierarchy of RLR regulation.

As EMCV infection promoted glucose influx in *Ubxn9*^+/+^ myocytes to levels equivalent in *Ubxn9*^−/−^ cells before and after infection (Fig. 3g), we postulated that EMCV activates UBXN9-controlled GLUT4 trafficking and glucose uptake. Confocal microscopy revealed GLUT4 translocation to the plasma membrane increased following EMCV infection in WT cells, whereas it remained constitutively elevated on the surface in *Ubxn9*^−/−^ myocytes and unaffected by EMCV infection, mirroring the kinetics of glucose uptake in these cells (Fig. 3g-i). The localization of GLUT4 on the surface was confirmed by using insulin treatment (positive control, Supplemental Fig. 7a, d), *Slc2a4*^−/−^ myocytes (negative control, Fig. 3h, i, Supplemental Fig. 7c, d) and the plasma membrane marker ZO-1 (Supplemental Fig. 7e). Consistent with these findings, siRNA knockdown of *Slc2a4* (GLUT4) in *Ubxn9*^−/−^ cells fully rescued IFN-β expression to WT levels after 3p-hpRNA treatment (Fig. 3j). Thus, these data reinforced the hypothesis that UBXN9 positively regulates RLR signaling through GLUT4 (Fig. 3a, f, j) and the activation of RLRs (via 3p-hpRNA or VSV/EMCV infection) can trigger GLUT4 exocytosis (Supplemental Fig. 7f). Notably, GLUT4-deficient myocytes continuously produced more IFN-β protein than their *Slc2a4*^+/+^ counterparts in all sugar conditions albeit lactate levels remaining comparable between the two cell types (Fig. 3k, l). Lastly, blockage of glucose uptake with Fasentin ^32^ failed to rescue the *Ifnb1* levels in EMCV-infected *Ubxn9*^−/−^ cells (Supplemental Fig. 6g, h). In total, these findings propose that the activation of RLRs induces GLUT4 translocation and UBXN9 strengthens the IFN-β response in a GLUT4-dependent manner that is uncoupled from glucose influx and lactate production.

### GLUT4 tethers RLRs to the plasma membrane

We demonstrated that TBK1 and IRF3 phosphorylation was affected in *Ubxn9*^−/−^ or *Slc2a4*^−/−^ cells (Fig. 1d, e, Supplemental Fig. 4e, f), suggesting the UBXN9-GLUT4 axis targets a step at or upstream of TBK1. To pinpoint the mechanism of GLUT4-regulated RLR signaling, we assessed the direct RLR-MAVS interaction as well as MAVS filament formation—the two immediate upstream events that are necessary for TBK1 activation–from WT and *Ubxn9*^−/−^ myocytes after 3p-hpRNA stimulation. Compared to WT control cells, *Ubxn9*^−/−^ myocytes had reduced binding of RIG-I to MAVS, which compromised MAVS oligomerization following 3p-hpRNA treatment (Fig. 3m, n). These data suggested that UBXN9-GLUT4 targets the earliest step of RLR pathways.

**Figure 4:**
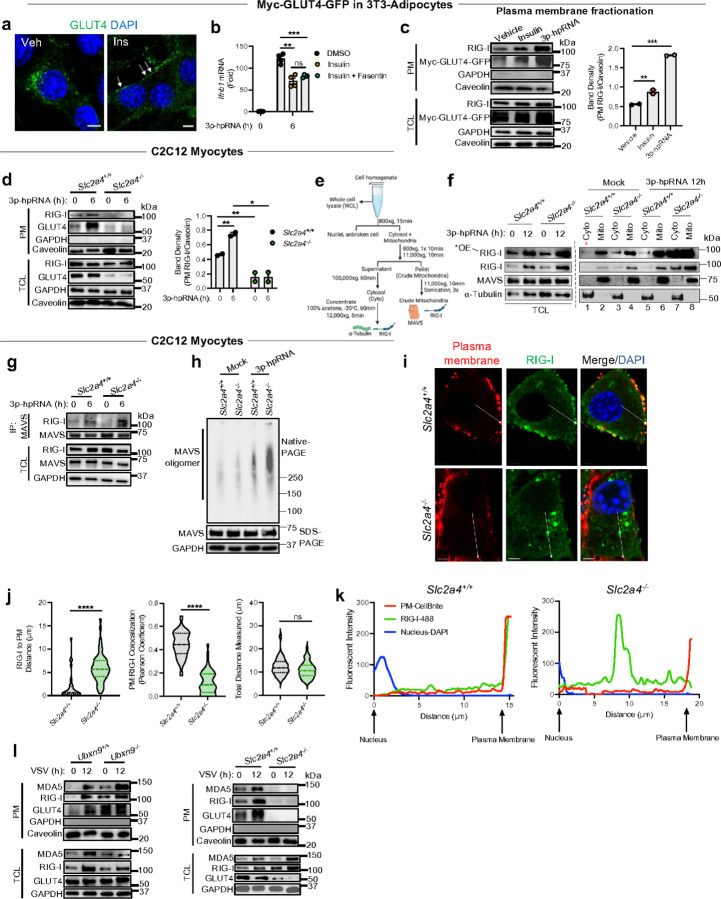
GLUT4 tethers RIG-I to the plasma membrane. **a,** confocal microscopic images of 3T3-L1 adipocytes stably expressing Myc-GLUT4-GFP either mock or insulin-stimulated (200 nM, 10 min). Scale bars, 5 μm. Arrows denote GFP signal (GLUT4) on the surface of cells after insulin. **b,** The *Ifnb1* mRNA in 3T3-L1 Myc-GLUT4-GFP adipocytes (n=3–4 biological replicates/group) that were treated simultaneously with insulin or insulin + Fasentin (100 μM) and transfected with 3p-hpRNA for 6 hr. **c,** immunoblot of the plasma membrane (PM) fraction and total cell lysate (TCL) proteins from 3T3-L1 Myc-GLUT4-GFP adipocytes that were either mock treated (vehicle), stimulated with insulin for 20 min or transfected with 3p-hpRNA for 6 hr (left panel). Protein band ratio of RIG-I to caveolin in the PM compartment of adipocytes (right panel). **d,** immunoblot of indicated proteins in the PM and TCL from *Slc2a4*^+/+^ and *Slc2a4*^−/−^ myocytes before and after 3p-hpRNA for 6 hr (left panel). Protein band ratio of RIG-I to caveolin in the PM (right panel). **e,** differential centrifugation protocol used to isolate TCL, crude mitochondria (Mito) and cytosolic (Cyto) fractions from C2C12 cells treated as in (**f**). **f,** distribution of various compartmental marker proteins from *Slc2a4*^+/+^ and *Slc2a4*^−/−^ cells subjected to subcellular fractionation as in (**e**) before (mock) and after 3p-hpRNA treatment for 12 hr. *OE, overexposure of both TCL and fractionation membranes to detect low RIG-I protein in cytosolic fractions. Red star (*) denotes low, but detectable RIG-I expression in cytosolic fraction of WT cells at steady state (mock, lane 1). **g,** endogenous MAVS co-immunoprecipitation (IP) from *Slc2a4*^+/+^ and *Slc2a4*^−/−^ C2C12 cells stimulated with 3p-hpRNA for 6 hr. **h,** MAVS filament formation in *Slc2a4*^+/+^ and *Slc2a4*^−/−^ cells stimulated with 3p-hpRNA for 6 h. **i,** confocal microscopic images of *Slc2a4*^+/+^ and *Slc2a4*^−/−^ C2C12 cells treated with 3p-hpRNA for 6 hr. Arrows track from the edge of nucleus to the edge of plasma membrane surface. Scale bar, 5 μm. **j, k,** distance between RIG-I and PM fluorescent signals (N=33–57 fields/group), RIG-I-PM colocalization (N=23–42 fields/group) (**j**), and a representative graph of the spatial distribution of fluorescence intensities in C2C12 cells treated with 3p-hpRNA for 6 hr as in (**i**) and shown in (**j**). **l,** immunoblot of indicated proteins in the PM fraction and TCL from *Ubxn9*^+/+^ and *Ubxn9*^−/−^ (left panel) and *Slc2a4*^+/+^ and *Slc2a4*^−/−^ myocytes (right panel) before and after VSV infection (MOI: 0.5) for 12 hr. Each knockout cell was paired with its respective WT clone for comparison. The results are representative of 2–3 independent experiments. Bar: mean ± SEM.*p<0.05, **p<0.01, ***p<0.001, ****p<0.0001 by one-way ANOVA for (**b, c**) or unpaired Student’s *t-*test (**d, j**).

As RLR signaling was suppressed when UBXN9 was deleted, we hypothesized that relocation of GLUT4 to the plasma membrane could negatively impact RLR signaling independent of glycolysis. To overcome the limitation of intracellular cytoskeleton staining with endogenous GLUT4 antibodies (Fig. 3h, Supplemental Fig. 7a-e) ^33^, we utilized the 3T3-L1 fibroblast/adipocyte cell line with a stably expressed myc-GLUT4-GFP reporter ^34^ that recapitulate the trafficking kinetics of endogenous GLUT4 in response to insulin (Fig. 4a). Further, these cells i) model adipocytes as important sources of IFNs during dysglycemia ^35^, and ii) are sensitive to RLR ligands ^36^. At steady state, GLUT4 was concentrated near the nucleus—consistent with its sequestration in GSVs at the Golgi/ERGIC ^34^—but acute insulin treatment (*t* = 10min) rapidly mobilized GLUT4 to the cell membrane, which suppressed the 3p-hpRNA-induced IFN-β response when compared with vehicle-treated cells (Fig. 4a, b). Moreover, blocking glucose uptake with Fasentin ^32^ did not rescue the insulin-mediated suppression (Fig. 4b). Although this data supports the postulate that GLUT4 suppresses RLR signaling independently of glucose transport (Fig. 3k, Supplemental Fig. 6g, h), it is unclear if GLUT4 physically impedes RIG-I.

As UBXN9-GLUT4 regulates RLR signaling likely at the level of receptors (Fig. 3m, n), we sought to visualize the spatial distribution of RIG-I when GLUT4 was mobilized to the cell surface. To focus on the primary RLR response, we chose an early timepoint (6hr after RLR activation) before RLR expression is amplified by the JAK-STAT1/2 pathway. Physiologically, RLR signaling is indispensable for the control of RNA viruses at the early stage of infection. Insulin or 3p-hpRNA (to promote GLUT4 translocation) induced significantly more RIG-I into the plasma membrane compartment (containing caveolin, a specific marker of the plasma membrane) than cells treated with vehicle alone (Fig. 4c, (Supplemental Fig. 7a-c, f). Accordingly, the pool of RIG-I and GLUT4 associated with the plasma membrane was enhanced in *Slc2a4*^+/+^ cells after 3p-hpRNA treatment, whereas RIG-I was nearly undetectable in this fraction from *Slc2a4*^−/−^ myocytes, suggesting the relocation of RIG-I to the plasma membrane is dependent on GLUT4 (Fig. 4d).

RIG-I expression in skeletal muscle is very low ^37^. To address if GLUT4 could hinder the rapidity of the primary IFN response through RLR tethering, we fractionated the crude mitochondria (marked by MAVS) and cytosolic fractions (marked by tubulin) from myocytes and assessed the spatial residency of RIG-I before and after 3p-hpRNA treatment ^38^ (Fig. 4e). Indeed, the cytosolic and crude mitochondrial pools of RIG-I were increased in *Slc2a4*^−/−^ cells (lane 7–8) as compared to their WT counterparts, which had significantly lower abundance before and after RLR stimulation (Fig. 4f, red star, lanes 1–2, 5–6). In the absence of treatment, deletion of GLUT4 also augmented the quantity of RIG-I in the mitochondrial fraction (Fig. 4f), consistent with previous reports of low, but detectable RIG-I in the crude mitochondria at steady state ^38^. These data suggest that GLUT4 abundancy in myocytes is sufficient to significantly deplete the cytosolic RIG-I pool accessible to MAVS before and after activation. These findings were further recapitulated at early timepoints in skeletal muscle cells and 3T3-L1 myc-GLUT4-GFP adipocytes (Supplemental Fig. 7h). Aligning with these fractionation results, the RIG-I-MAVS interaction and MAVS filament formation were enhanced in *Slc2a4*^−/−^ cells only after 3p-hpRNA treatment relative to control myocytes (Fig. 4g, h). Confocal microscopy further showed RIG-I juxtaposed plasma membrane signals in *Slc2a4*^+/+^ cells, whereas bright, segregated RIG-I puncta were observed in the perinuclear area of *Slc2a4*^−/−^ myocytes, consistent with active signaling and a magnified pool of cytosolic RIG-I that was untethered by GLUT4 ^39^ (Fig. 4f, i, Supplemental Fig. 7g, h). Measuring the distance of RIG-I to the plasma membrane indicated that deletion of GLUT4 significantly dissociated these two fluorescent signals from a near overlap in *Slc2a4*^+/+^ (mean = ~ 1.8µm) (Fig. 4i-k). In total, these findings, in conjunction with the induction of IFN-β (Fig. 2), demonstrate that the GLUT4 governance of RIG-I redistribution to the plasma membrane profoundly influences the primary RLR signaling program.

The magnitude of GLUT4 mobilization is directly proportional to the total UBXN9-GLUT4 complexes that are dissociated during insulin treatment ^16^. The results in Fig. 3 indicated deletion of UBXN9 accelerates GLUT4 trafficking (Fig. 3h, Supplemental Fig. 7b, d). We further tested the specificity of RIG-I relocation by tracking the cellular localization and distribution of GLUT4 and RLRs in tandem during virus infection. Despite RIG-I being the primary sensor of VSV, both RIG-I and MDA5 increased simultaneously with GLUT4 in the plasma membrane following VSV infection in WT cells, indicating viral RNA-induced conformational changes in these sensors is not a prerequisite for GLUT4-mediated tethering (Fig. 4l). In contrast, RLRs trafficked in tandem with the constitutively translocated pool of GLUT4 in *Ubxn9*^−/−^ cells, which was virtually eliminated from the plasma membrane when GLUT4 was deleted (Fig. 4l). The synchronized translocation of RLRs with GLUT4 mirrored the kinetics of glucose uptake in WT, *Ubxn9*^−/−^ and *Slc2a4*^−/−^ cells before and after insulin treatment (Supplemental Fig. 7i, j). Overall, these results support our hypothesis that, within the hierarchy of RLR regulation, GLUT4-mediated redistribution of RIG-I to the plasma membrane is a dominant mechanism in skeletal muscle and adipocytes.

### GLUT4 binds RLRs and translocates them to the plasma membrane to curb RLR signaling

**Figure 5:**
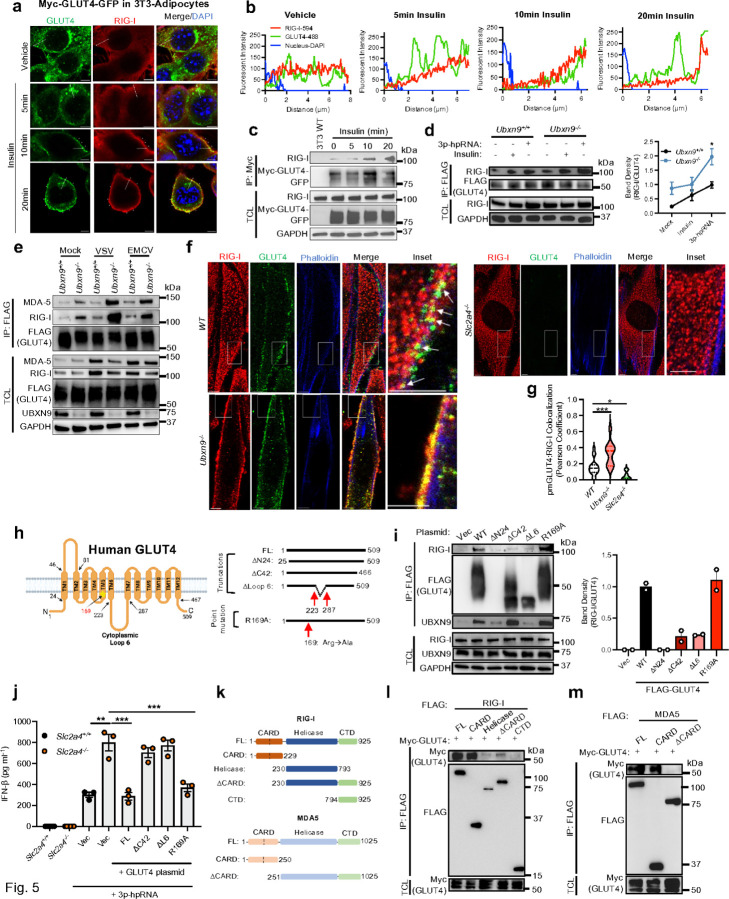
GLUT4 binds RLRs and translocates them to the plasma membrane to curb RLR signaling **a,** confocal microscopic images of 3T3-L1 Myc-GLUT4-GFP adipocytes that were either mock treated or stimulated with insulin (200 nM) for 5, 10 or 20 min. Arrow heads denote the colocalization of GLUT4 and RIG-I on the cell surface. **b,** representative graphs of the spatial distribution of DAPI, RIG-I and GLUT4 fluorescence intensities along the vector arrow from the edge of the nucleus to the cell surface in (**a**). Scale bar, 10 μm. Vehicle, media only without insulin. **c,** co-IP from 3T3-L1 Myc-GLUT4-GFP adipocytes stimulated with insulin for various times. 3T3 WT without Myc-GLUT4 serves as a negative control for IP. **d,** co-IP of FLAG-GLUT4 and endogenous RIG-I from *Ubxn9*^+/+^ and *Ubxn9*^−/−^ C2C12 cells transfected with FLAG-GLUT4 for 24 hr and either mock treated, stimulated with insulin for 20 min or stimulated with 3p-hpRNA for 12 hr. Quantification of protein band ratios of RIG-I/GLUT4 in IP elution samples from C2C12 cells stimulated with insulin or 3p-hpRNA (right panel). **e,** co-IP of FLAG-GLUT4 and endogenous RLRs from *Ubxn9*^+/+^ and *Ubxn9*^−/−^ C2C12 that were transfected as in (**d**) and then infected with VSV or EMCV for 12 hr. EMCV and VSV, MOI: 0.5. **f,** confocal microscopic images of *WT*, *Ubxn9*^−/−^ and *Slc2a4*^−/−^ C2C12 myocytes infected with VSV for 12h (MOI: 0.5). Inset is higher magnification derived from ROI box in lower magnification image. Scale bar, 5 μm. White arrows denote colocalization of GLUT4 and RIG-I on the plasma membrane in WT cells. **g,** plasma membrane GLUT4 (pmGLUT4) and RIG-I colocalization in *WT*, *Ubxn9*^−/−^ and *Slc2a4*^−/−^ C2C12 myocytes (N=10–19 fields/group) infected as in (**f**). **h,** schematic diagram of human GLUT4 membrane topology (left panel) and deletion/point mutant constructs (right panel). Yellow star (169, red text) denotes point mutation of glucose binding residue. **i,** co-IP of the truncated forms of GLUT4 (**h**) with endogenous RIG-I and UBXN9 from HEK293T cells. Quantification of protein band ratios of RIG-I to GLUT4 in IP elution samples (right panel). Vector, pcDNA3.1-FLAG. **j,** IFN-β protein concentrations from C2C12 cells transfected with WT or mutant GLUT4 plasmids for 24 h and then either mock treated or stimulated with 3p-hpRNA for 12 hr. **k,** schematic representation of the domain structure of RIG-I and MDA5 and its truncation mutants used for co-IP experiments in (**l, m**). FL, full length; CARD, caspase activation and recruitment domain; ∆CARD, RIG-I with deletion of CARD domain; CTD, C-terminal domain. **l, m,** co-IP of the WT and truncated mutants of RIG-I (**l**) or MDA5 (**m**) with GLUT4 in HEK293T cells. The results are representative of 2–3 independent experiments. Bar: mean ± SEM. *p<0.05, **p<0.01, ***p<0.001 by one-way ANOVA (**d, j**) or unpaired Student’s *t*-test (**g**).

The above findings suggest that trafficking of GLUT4 to the plasma membrane suppresses RLR signaling by repositioning RIG-I away from MAVS. Mobilization of GSVs involves the vesicular trafficking machinery as well as numerous accessory proteins in close association with GLUT4 ^16, 40^. Considering other protein(s) of anterograde transport could be responsible for RLR tethering, we first examined the interaction between GLUT4 and RLRs under the same conditions known to promote GLUT4 translocation (Figs. 3 and 4, Supplemental Fig. 7). Using 3T3-L1 myc-GLUT4-GFP adipocytes, line tracing demonstrated a shift in GLUT4 and RIG-I signals from a uniform distribution at steady state (Vehicle), to the cell periphery after insulin treatment (Fig. 5a, b). Moreover, the GLUT4-RIG-I interaction strengthened over time upon stimulation with insulin (in adipocytes and C2C12 myocytes), 3p-hpRNA and virus infection in WT myocytes (Fig. 5c-e). Intriguingly, even in the face of constitutive GLUT4 trafficking and RLR tethering (Fig. 4l, Fig. 3h, Supplemental Fig. 7b, i, j), 3p-hpRNA and virus infection continued to induce stronger binding between GLUT4 and RLRs in *Ubxn9*^−/−^ cells (Fig. 5d, e). Confocal microscopy confirmed extensive colocalization of endogenous GLUT4 with RIG-I on the plasma membrane after VSV infection in *Ubxn9*^−/−^ as compared to WT cells (Fig. 5f, g, inset images). Such subcellular relocation of RIG-I to the membrane was dependent on GLUT4 as the brightest RIG-I signals were concentrated near the perinuclear region and failed to colocalize with GLUT4 or the plasma membrane in *Slc2a4*^−/−^ cells (Fig. 5f, g). Lastly, we detected equivalent RLR tethering by ectopic GLUT4 in *Ldha*^+/+^ and *Ldha*^−/−^ cells after 3p-hpRNA treatment, indicating this mechanism operates upstream of lactate inhibition (Supplemental Fig. 8a, b). Thus, GLUT4 directly sequesters RLRs during translocation to the plasma membrane, induced by either exogenous stimuli or after UBXN9 deletion

All GLUT proteins possess 12 transmembrane segments which facilitate their incorporation into the plasma membrane ^13^ (Fig. 5h). GLUT4 has a 24 amino acid (aa) long cytoplasmic N-terminus, a large cytoplasmic loop (Loop 6, ~ 65 aa) that is bound by UBXN9 to sequester it at the Golgi/ ERGIC ^34^, and a 42 aa-long cytoplasmic C terminus. The remaining residues are in short, 8–12 aa intracellular loops (Loop 2, 4, 8 and 10). Therefore, given RLRs reside in the cytoplasm, we reasoned that the N-, C-terminus or Loop 6 of GLUT4 might mediate its interaction with RLRs. We generated three FLAG-tagged deletion mutant constructs of GLUT4: ΔN24, ΔC42 and ΔL6, and included a point mutation, Arg169Ala (R169A), that disrupts glucose uptake by > 70% ^41^ (Fig. 5h). Although the R169A mutant failed to rescue glucose uptake relative to *Slc2a4*^−/−^ cells reconstituted with WT GLUT4, it phenocopied the RIG-I binding potential of the WT protein (Supplemental Fig. 8d, e). These data reinforced the hypothesis that the transport activity of GLUT4 is decoupled from its tethering of RLRs to the plasma membrane. All the GLUT4 mutants were expressed except for ΔN24, which was likely unstable due to misfolding, among many other possibilities (Fig. 5i). Importantly, the ΔC42 and ΔL6 mutants were unable to bind RIG-I compared to FL and R169A GLUT4 (Fig. 5i). Further co-immunoprecipitation from these constructs revealed deletion of loop 6 compromised the UBXN9 interaction (Fig. 5i), which is the competitive binding site between RIG-I and UBXN9 (Supplemental Fig. 8f). Functionally, reconstitution of *Slc2a4*^−/−^ cells with the ΔC42 and ΔL6 mutants failed to inhibit RLR signaling to the levels in *Slc2a4*^+/+^ cells, whereas FL WT and R169A GLUT4 inhibited 3p-hpRNA-induced IFN-β expression (Fig. 5j). These findings indicated that the C-terminus and Loop 6 of GLUT4 are necessary for binding and suppressing RLRs.

Finally, to address where GLUT4 bound to RIG-I, we generated four domain mutants of RIG-I (FLAG-tagged): caspase activation and recruitment domain (CARD), helicase, ΔCARD and the C-terminal domain (CTD) and assessed their reciprocal binding efficiencies to GLUT4 (Myc-tagged) (Fig. 5k). The FL and RIG-I CARD domain alone strongly interacted with GLUT4, whereas the helicase and the CTD failed to do so (Fig. 5l, Supplemental Fig. 8c). Similarly, the MDA5 FL protein and CARD domain was sufficient for GLUT4 binding (Fig. 5m). Importantly, failure of the ΔCARD mutants to bind GLUT4 established this domain as the putative binding site between RLRs and GLUT4 (Fig. 5l, m). In summary, GLUT4 directly sequesters RIG-I and MDA5 to the plasma membrane during trafficking and blunts the primary activation of RLRs by interacting with the CARD domain.

### Promotion of UBXN9 cleavage and GSV release underlie RLR sequestration during virus infection

**Figure 6:**
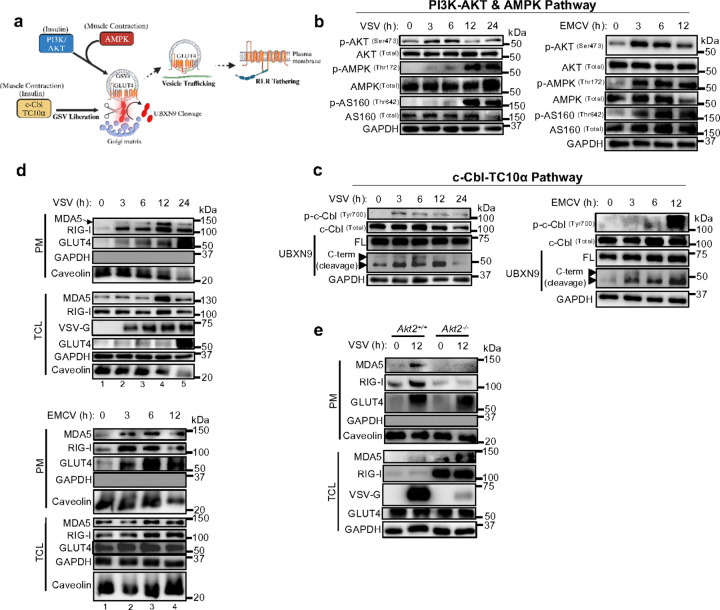
Promotion of UBXN9 cleavage and GSV release underlie RLR sequestration during virus infection. **a,** simplified schematic of the converging pathways that act on GLUT4 storage vesicle (GSV) trafficking to promote RLR tethering. **b, c,** immunoblot of indicated proteins in the total cell lysate (TCL) from WT C2C12 cells before and after VSV (MOI: 0.5) (left panel) or EMCV (MOI: 0.5) infection (right panel). **d,** immunoblot of indicated proteins in the plasma membrane (PM) fraction and TCL from WT C2C12 myocytes infected as in (**b, c**) with VSV (upper panel) or EMCV (lower panel). **e,** immunoblot of indicated proteins in the PM fraction and TCL from *Akt2*^+/+^ and *Akt2*^−/−^ myocytes before and after VSV infection (MOI: 0.5) for 12 hr. Mock, medium alone without virus. The results are representative of 2–3 independent experiments.

Virus-induced metabolic reprogramming can be attributed to, in part, manipulation of upstream enzymes in various glycolytic pathways ^7, 8, 42^. Our results suggest that in addition to RNA virus infection (Fig. 3h, 4l), acute insulin signaling can promote RLR tethering (Fig. 4c, 5a-d). Therefore, we focused on the conserved upstream nodes in the PI3K-AKT, AMPK and c-Cbl-TC10a GLUT4 trafficking pathways that may contribute to RLR sequestration (Fig. 6a). We detected an acute increase in AKT phosphorylation at 3–6 hpi that returned to the baseline shortly thereafter (Fig. 6b). On the other hand, AMPK activation was gradual and sustained throughout the course of RNA virus infection (Fig. 6b). Importantly, microtubule-based movement of GSVs was activated reflected by the phosphorylation of AS160 (Tbc1D4) (Fig. 6b).

The c-Cbl pathway culminates in cleavage of UBXN9 to liberate GSVs from the Golgi matrix—accordingly, and consistent with endoproteolytic processing of UBXN9 after insulin treatment ^19, 43^, virus infection led to a noticeable increase in c-Cbl phosphorylation and the production of UBXN9 C-terminal cleavage products (Fig. 6c). Thus, virus infection coordinates GLUT4 translocation through the activation of vesicle trafficking and cleavage of UBXN9.

We further tested whether the activation of GLUT4 pathways was synchronized with the relocation of RLRs to the surface during virus infection. Indeed, GLUT4 and RLRs were transported to the plasma membrane in unison, although the speed at which GLUT4 trafficked differed depending on the RNA virus (Fig. 6d). Agreeing with our fractionation results (Fig. 4f), the peak of RLR sequestration (lane 4, VSV; lane 3, EMCV) was preceded by the rapid relocation of steady state RIG-I and MDA5 (lane 1–3, VSV; lane 1–2, EMCV) (Fig. 6d). As the infection progressed, even in the presence of more GLUT4, a significant drop in RLR surface abundance was evident at later timepoints (lane 5, VSV; lane 4, EMCV), A similar phenomena was also noted in 3T3-L1 myc-GLUT4-GFP reporter cells as early as 60 minutes after insulin stimulation (Supplemental Fig. 9a), suggesting confinement is a transient event and RLRs are eventually relinquished from GLUT4-mediated tethering.

As AKT regulates GLUT4 trafficking, we hypothesized that the relocation of RLRs during virus infection is dependent on AKT. We next genetically deleted the dominant Akt isoform in skeletal muscle, *Akt2* ^44^, and evaluated GLUT4 trafficking after RNA virus infection (Supplemental Fig. 9b). In line with previous reports ^44^, *Akt2*^−/−^ cells had comparable translocation of GLUT4 to the surface after VSV and EMCV infections, relative to *Akt2*^+/+^ controls (Fig. 6e, Supplemental Fig. 9c). Loss of Akt2 may have been compensated for by the sustained activation of AMPK (Fig. 6b), which phosphorylates similar AS160 residues as AKT ^45^. Despite equivalent GLUT4 trafficking, *Akt2*^−/−^ cells were unable to mobilize RIG-I and MDA5 to the plasma membrane fraction, while WT cells showed a consistent increase in RLR abundance after virus infection (Fig. 6e). Moreover, *Akt2*^−/−^ had elevated RLR expression before and after VSV infection relative to those in the WT cells; these heightened ISGs in *Akt2*^−/−^ likely afforded better protection from VSV infection (Fig. 6e). In total, these data revealed AKT2 is dispensable for GLUT4 translocation yet necessary for sequestration of RLRs to the plasma membrane.

### Myopathic diseases are associated with decreased GLUT4 expressions and elevated interferon signatures in skeletal muscle

**Figure 7:**
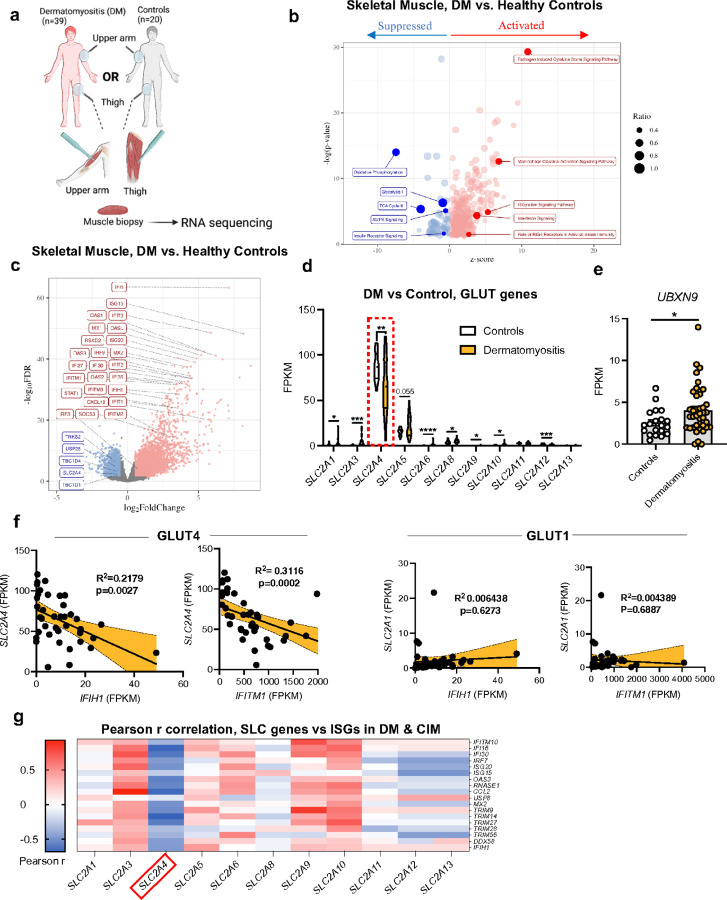
Myopathic diseases are associated with decreased GLUT4 expression and elevated interferon signatures in skeletal muscle. **a,** schematic overview of dermatomyositis (DM) study (GSE143323) from which the data in (**b-f**) are derived. N=39 DM and 20 healthy controls. **b,** Ingenuity Pathway Analysis (IPA) for pathways activated or suppressed in DM patients compared to healthy controls. The Log P value and Z-score represent significance and activity of pathway enrichments (z-score > 0, activated; z-score < 0, inhibited), respectively. The dot size represents the number of genes found enriched/suppressed compared to the whole gene set in that pathway (ratio). Highly upregulated pathways related to interferon signaling and highly downregulated pathways associated with metabolism are denoted in boxes and filled with their respective signature colors. c,volcano plot depicting differentially expressed genes (DEGs) (FDR <0.05 and Log2FC >0.5) between healthy controls and DM patients. DEGs with a fold change >0.5 are indicated in red; DEGs with a fold change <0.5 are in blue. Nonsignificant DEGs are indicated in grey. FDR, false discovery rate. **d, e,** relative FPKM of *SLC2* genes (GLUTs) (**d**) and *UBXN9* (**e**) in DM patients compared to controls. Dotted red box highlights expression differences for *SLC2A4* (GLUT4). **f,** simple linear regression analysis of *SLC2A4* (GLUT4) or *SLC2A1*(GLUT1) FPKMs with *IFIH1* and *IFITM1* FPKMs from DM patients. **g,** representative heatmap demonstrating correlations between all *SLC2A*(GLUTs) and ISGs identified to be upregulated in both DM and critical illness myopathy (CIM) study subjects (Supplemental Fig. 10C), calculated by Pearson r correlation. Red box highlights a significant negative correlation of GLUT4 with all ISGs. Bar: mean ± SEM. *p<0.05, **p<0.01, by Mann-Whitney test in (**d, e**) or Pearson r correlation (**g**).

The experiments above revealed a non-canonical function of GLUT4 in the relocation of RLRs, which perturbs signal transduction and antiviral immune responses. To test the biomedical impact of the putative tethering mechanism of GLUT4, we analyzed two datasets of skeletal muscle tissue biopsied and sequenced directly from patients with critical illness myopathy (CIM) (PRJNA491748) or dermatomyositis (DM) (GSE143323) (Fig. 7a, Supplemental Fig. 10a) ^46, 47^. Idiopathic inflammatory myopathies are autoimmune conditions characterized by muscle inflammation and weakness ^48^, enriched inflammatory gene signatures, and an upregulation of RIG-I that is now considered a biomarker of DM ^49^.To first investigate the pertinence of GLUT4 in these autoimmune disorders, we analyzed the pathways enriched in DM patients compared to healthy skeletal muscle using QIAGEN ingenuity pathway analysis (IPA) ^46^. Of the 382 pathways upregulated in DM muscle, IFN-related pathways were among the most enriched including ISGylation Signaling, Interferon Signaling and RIG-I Receptors in Antiviral Immunity (Fig. 7b). Consistent with previous reports, we also observed Pathogen Induced Cytokine Storm Signaling—comprised of inflammatory and interferon genes—was the most significantly activated pathway in myopathic patients by p-value and Z-score ^50^. Similar inflammatory pathways were also enriched in the muscle of CIM patients compared to controls (Supplementary Fig. 10b; red dots). Of note, Oxidative Phosphorylation, Glycolysis, TCA cycle, and AMPK Signaling pathways were downregulated in DM patients, indicating a general dysregulation of muscle metabolism (Fig. 7b). Further RNA-seq analysis of the same muscle comparing the log_2_ fold change in genes (FDR < 0.05) revealed similar patterns of exaggerated interferon/inflammatory transcripts in the primary (*IFIH1, IRF3)* and secondary (*STAT1*, *MX1, ISG15*) RLR response in myopathic patients (Fig. 7c). These results are consistent with previously published datasets reporting heightened interferon pathways in myopathic skeletal muscle ^47,51, 52^. Unexpectedly, *SLC2A4* (encoding GLUT4), and several genes involved in GLUT4 trafficking ^16^, were downregulated in DM muscle relative to controls (Fig. 7c, Supplemental Fig. 10d).

Since several GLUTs could contribute to the dysregulation of metabolic pathways in myopathic muscle, we next compared the expression of all the detected *SLC2A* genes in DM patients. Notably, only *SLC2A4* transcripts were significantly decreased in myopathic muscle (Fig. 7d, dotted box), whereas several other *SLC2A* transporters and UBXN9 were increased, potentially reflecting a compensation in glucose homeostasis in DM (Fig. 7d, e). Further, *SLC2A4* represented the only DEG of the *SLC2A* transporter family in CIM patients (Supplemental Fig. 10c). Linear regression analysis of relative GLUT4 expression with ISGs on a patient-by-patient basis revealed that GLUT4 inversely correlated with *IFIH1*, *IFITM1*, *DDX58* and *OAS1A* (Fig. 7f, Supplemental Fig. 10e). Pearson’s correlation analysis indicated GLUT4 was the only transporter that negatively correlated with a common set of IFN genes upregulated in both datasets (Fig. 7g). Importantly, neither GLUT1 expression from DM patients (the other major glucose transporter in muscle) nor GLUT4 from healthy muscle demonstrated such a relationship (Fig. 7f, g, Supplemental Fig. 10e). Collectively, these data highlight a key relationship between lower GLUT4 levels and heightened IFN signatures in patients with various myopathic diseases.

## DISCUSSION

GLUT4 is the dominant, insulin/muscle contraction-regulated glucose transporter in adipose and muscle tissues, and indispensable for the maintenance of whole-body glucose homeostasis. Despite this, and the previous literature that GLUTs support immune activation, GLUT4 has not been thoroughly investigated in the context of innate immunity. Here, we demonstrate that GLUT4 inhibits the major cytosolic viral RNA-sensing pathways, RIG-I and MDA5, by confining RLRs to the plasma membrane, displacing them from their downstream adaptor MAVS and attenuating the primary antiviral type I IFN response. This conclusion is further supported by studies with UBXN9, an important tether of GLUT4. Deletion of UBXN9 leads to continuous trafficking of GLUT4 to the plasma membrane (comparable to the effect of acute insulin stimulation) ^17, 18^, together with redistribution of the resident pool of RLRs to the surface, and suppression of antiviral immune responses during RNA virus infection. Mechanistically, GLUT4-mediated tethering of RLRs was mediated by binding of the large cytoplasmic loop (L6) and C-terminus of GLUT4 to the CARD domain of RLRs. The ΔC42 and ΔL6 mutants also failed to restore normal signaling to *Slc2a4*^−/−^ cells, suggesting both domains contribute to the sequestration and inhibition of RLRs. These results are concordant with the idea that only UBXN9-free GLUT4 on the plasma membrane is able to tether RLRs as UBXN9 occupies the Loop 6 of GLUT4 at the Golgi matrix ^18^. Thus, the compartmentalization of RNA sensors by translocated GLUT4 represents an important event upstream of RLR activation and an additional regulatory mechanism of innate immune signaling.

A growing body of evidence has highlighted that SLCs can have, beyond their established roles as transporters, functions related to the trafficking and signaling of innate immune receptors ^53^, now termed “transceptors” (transporter-receptor). For example, the histidine-peptide cotransporter SLC15A4 controls TLR7,9-induced cytokine responses independent of its ligand-binding potential ^54^. Moreover, the transporter activity of GLUT3 is dispensable for the coordination of IL-4/STAT6 signaling in M2 macrophage polarization ^55^. Our work aligns with this transceptor model, suggesting a transporter-independent role of GLUT4 in RLR signaling. This proposal is substantiated by four pieces of evidence: (1) mutation of the glucose-binding residue (R169A) interacted equally with RLRs as compared to WT GLUT4, (2) pharmacological inhibition of glucose uptake failed to rescue the GLUT4-mediated suppression of IFN production in adipocytes and skeletal muscle, (3) glucose-free culture conditions did not alter the *Slc2a4*^−/−^ phenotype during RLR signaling, and (4) deletion of GLUT4 did not abolish steady state glucose uptake, but effectively depleted the insulin- and virus-responsive pool critical for relocating RLRs to the surface. Although glucose uptake by GLUT4 is not involved in RLR signaling, we and others ^9, 10^ have demonstrated RNA virus infection can promote glucose influx, providing a rapid source of ATP and raw materials for virus replication ^56^. With these data in mind, our study cannot exclude the role of other immunomodulating metabolites, such as itaconate (and its derivatives), which feedback on STAT1 to downregulate IFN-β responses ^57^. This metabolic change is generated during the shift to glycolysis and therefore warrants future investigation during RLR activation.

Investigation into virus hijacking of GLUT4 trafficking revealed the activation of AKT, AMPK and c-Cbl, aligning with numerous reports that viruses exploit upstream enzymes in glucose sensing pathways to support replication ^8, 58 42^. However, our work highlights the apparent dichotomy between GLUT4 translocation and RLR confinement. While the proper delivery of GLUT4 to the surface is organized by mechanisms unique to adipocytes and skeletal muscle cells—including the phosphorylation of AS160 by AKT and AMPK ^45, 59^—RLR tethering was nearly abrogated in *Akt2*^−/−^ cells despite intact GLUT4 translocation. These data argue that GLUT4 is necessary but not sufficient for RLR sequestration, and AKT2 may directly catalyze this interaction. Evidence for this postulate further stems from our studies in *Ubxn9*^−/−^ cells whereby constitutive GLUT4 trafficking continued to strengthen its interaction with RLRs following subsequent AKT activation (e.g., insulin, virus infection). In fact, the eventual untethering of RLRs from GLUT4 was synchronized with the return of AKT to its pre-infection phosphorylation state. Given the conflicting reports of AKT2’s role for regulating GLUT4 translocation *in vivo* ^44^, and its suppression of 14–3-3 chaperones necessary for IFN-β responses ^60, 61, 62^, future studies aim to address the relative contribution of the two proposed functions for AKT2, *i.e*., conventional GLUT4 trafficking and the control of RLR signaling transduction.

The transient tethering of RLRs to the plasma membrane further coincided with cessation of IFN signaling as reflected by an overall decrease in RLR expression. Akin to canonical regulation of RLRs in the cytosol, post-translational modifications–such as ubiquitination–also terminate signaling of surface receptors by degrading signalosome components or mislocalizing sorting adapters from their subcellular site of signal transduction ^63^. Indeed, c-Cbl, which was phosphorylated after RNA virus infection herein, conjugated K48-linked polyubiquitination of RIG-I near the plasma membrane ^58^. As degradative polyubiquitin chains are linked at later stages of infection ^64^, sequestered RLRs may present an opportunity to couple compartmentalization mechanisms with degradative pathways.

Stringent regulation of PRRs is necessary to avoid recognition of self-ligands that elicit autoinflammatory conditions ^4 5^. One such mechanism is compartmentalization, which is best exemplified by shielding the ligand binding domains of endosomal-bound TLRs from cytoplasmic contents ^65^. Of note, compartmentalization is also applicable to the cytosolic DNA sensor, cGAS ^66, 67^. Our data offers further support of this idea, as GLUT4 attenuated the primary stage of signaling by sequestering the pool of preexisting (i.e., steady state and 3–6hpi) and *de novo* cytosolic RLRs (i.e., JAK/STAT-induced ISG) away from MAVS. These results align with recent reports that RLRs are proximally regulated by the actin cytoskeleton ^68, 69^. Notably, tethering RLRs is reliant on the cleavage of UBXN9 from GLUT4 and subsequent trafficking of GSVs, which is constitutive at a low level but induced following virus infection. As GSVs contain approximately five GLUT4 molecules per vesicle ^43, 70^, viral-induced UBXN9 cleavage further increases the likelihood of a GLUT4-RLR interaction. Furthermore, generation of this UBXN9 C-terminal cleavage product stimulates thermogenesis ^19^ and promotes a feed-forward circuit that could account for increased body temperature (i.e., fever) when the IFN-β response is attenuated by GLUT4.

**Figure 8:**
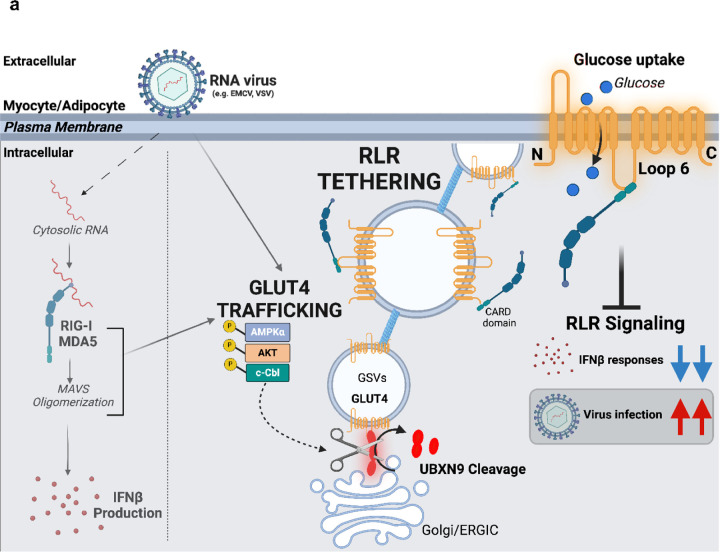
GLUT4 compartmentalizes RLRs to the plasma membrane to attenuate RLR signaling. Viral RNA is sensed by cytosolic RIG-I and MDA5 that leads to MAVS oligomerization at the mitochondria for IFN-β production. During viral infection, GLUT4 trafficking machinery are activated—including AKT, AMPK and c-Cbl—that promote UBXN9 cleavage, thus liberating GLUT4 storage vesicle (GSVs) for surface translocation. RLRs are then sequestered to the plasma membrane by trafficking GLUT4, leading to attenuated IFN-β responses and augmented virus replication. Of note, GLUT4 can effectively tether the steady state and IFN-β-induced pool of cytosolic RLRs. The loop 6 and C-terminus of GLUT4 is responsible for binding to the RLR CARD domain. Colored “P” circle denotes AKT, AMPK and c-Cbl are phosphorylated after virus infection. GLUT4 storage vesicles, GSVs. Mitochondrial antiviral-signaling protein, MAVS. Caspase activation and recruitment domains, CARDs. RIG-I-like receptor, RLR.

Beyond an immune evasion strategy, it is tempting to speculate upon the potential protection that is afforded by compartmentalizing RNA sensors to the plasma membrane. Under such a model is where our results may be the most relevant, as *GLUT4* and its associated trafficking genes (e.g., *TBC1D4/AS160*) are significantly downregulated in the muscle of dermatomyositis patients presenting with exaggerated interferon signatures. Overall, our data indicates that RLRs are poised for activation by virus infection when GLUT4 is largely sequestered by UBXN9. Progression of the response leads to the release of GLUT4 from UBXN9, which redistributes RLRs to the surface and attenuates RLR signaling and antiviral immunity (Fig. 8).

In sum, our study identifies spatial confinement as a dominant strategy to control RLR signal transduction in skeletal muscle and adipocytes and unveils a previously unknown glucose-independent function of GLUT4 in the regulation of innate immunity. Future studies are necessary to elucidate the molecular details of the GLUT4-RLR interaction at the plasma membrane, specifically how AKT2 promotes the sequestration of RLRs, when GLUT4 trafficking intervenes with signal transduction and the role of RLR signaling in metabolic and autoinflammatory diseases.

## MATERIALS AND METHODS

### Mouse models

All animal procedures were approved by the Institutional Animal Care and Use Committee at UConn Health adhering to federal and state laws. Mice with the exon 5 of Ubxn9 flanked by loxP sites (*Ubxn9*^*flox/flox*^) were generated via homologous recombination. The homozygous *Ubxn9*^*flox/flox*^ were then crossed with homozygous tamoxifen-inducible Cre recombinase-estrogen receptor T2 mice (The Jackson Laboratory, Stock # 008463; Rosa26^Cre-ERT2^) to generate male and female Cre^+/−^*Ubxn9*^*fl*^ littermates, which were mated to produce Cre^+/−^*Ubxn9b*^*fl/fl*^, *Ubxn9b*^*fl/fl*^, and Cre^+/−^. To induce global *Ubxn9* knockout in ≥ 5-week-old mice (*Ubxn9*^−/−^), 1mg of tamoxifen was administered (dissolved in corn oil) to each mouse every other day for a total of 5 injections. *ERT2-Cre*^+/−^*Ubxn9*^*fl/fl*^ treated with corn oil served as the wild-type control (*Ubxn9*^+/+^). Tamoxifen was allowed to be cleared 10 days after the last injection before experimentation. Both male and female mice were used between 7–18 weeks of age. Genotyping was performed with genomic DNA and Choice Taq Blue Mastermix (Denville Scientific, Cat# CB4065–8) using the following PCR protocol: 95°C for 1 s, 34 cycles of 94°C for 1 min, 60°C for 30 s, 72°C for 30 s, and then 72°C for 7 min, 4°C to stop. The genotyping primers for the loxP sites in exon 5 were: Ubxn9 F 5’GCTTCTCTCAAAGCTGGAGAGTCAC; R 5’ CAAGGCACTGGGCCAGGGAG. The PCR reaction resulted in a product of 226bp (WT) and/or 276 bp (loxP). The Cre primers were common: WT F 5’-AAGGGAGCTGCAGTGGAGTA; WT R 5’-CCGAAAATCTGTGGGAAGTC; mutant R 5’-CGGTTATTCAACTTGCACCA. The PCR reaction resulted in a product of 297 bp (WT) and/or 450bp (Cre).

The GLUT4 LoxP mice (*Slc2a4*^*flox/flox*^) were generated as previously described ^71^ and provided by Carol A. Witczak (Indiana University). *Slc2a4*^*fl/fl*^ mice were then bread with muscle creatine kinase Cre recombinase transgenic mice (The Jackson Laboratory, strain # 006475; MCK-Cre^+^). Heterozygote mice (*Slc2a4*^*fl/+*^-*MCK*^*Cre*^) were used for breeding to generate the following genotypes: wild-type (*Slc2a4*^+/+^), GLUT4 LoxP HET (*Slc2a4*^*fl/+*^), LoxP homozygous (*Slc2a4*^*fl/fl*^), MCK-Cre+ (control), muscle-specific GLUT4 heterozygous (*Slc2a4*^*fl/+*^ *MCK*^*Cre*^) and muscle-specific GLUT4 knockout (KO; *Slc2a4*^*fl/fl*^ *MCK*^*Cre*^). For primary myocyte studies, *Slc2a4*^*fl/fl*^ MCK^Cre^ (KO) and MCK-Cre+ (WT) mice were used at 12-weeks of age. For genotyping, the PCR protocol for GLUT4 LoxP was 95°C for 3min, 35 cycles of 95°C for 20sec, 64°C for 30sec, 72°C for 50sec, 72°C for 2min, 10°C to stop. The genotyping primers for LoxP were F: 5'GGCTGTGCCATCTTGATGACC 3’; R: 5'ACCCATGCCGACAATGAAGTTAC 3’. This PCR resulted in a WT band (~ 752 bp) and a GLUT4 LoxP + band (~ 900bp). The Cre primers for MCK-Cre were F: 5’TAAGTCTGAACCCGGTCTGC 3’; R: 5’GTGAAACAGCATTGCTGTCACTT 3’ and resulted in Cre + band (450bp). An internal control primer for DNA quality was also included F: 5'CAAATGTTGCTT GTCTGGTG 3’; R: 5'GTCAGTCGAGTGCACAGTTT 3’ and resulted in a band ~ 200bp. The Cre PCR protocol was 94°C for 2min, stepdown protocol of 0.5°C/cycle at 94°C for 1min, 65°C 30sec, 68°C 50sec, then 28 cycles of 94°C 1min, 60°C for 30sec, 72°C for 50sec, then 72°C for 7min, 10°C hold (The Jackson Lab strain # 006475; MCK-Cre^+^, Cre primer protocol).

### Reagents and antibodies

The anti-GAPDH (Cat # 60004–1-Ig, 1:2000) and anti-GFP (Cat # 50430–2-AP, 1:100 for IF) were purchased from Proteintech Group, Inc (Rosemont, IL). The mouse anti-Myc (Cat # TA150121, 1:1000) and mouse Slc2a4 siRNA duplex (Cat # SR416343, 10nM final concentration) were obtained from Origene Technologies, Inc (Rockville, MD). The rabbit anti-GLUT4 (Cat # PA5–23052, 1:1000), rabbit anti-MDA5 (Cat # 700360, 5µg/mL for IF) Hoescht 33342 solution (Cat # 62249, 1:10,000 for IF), Pierce Protein A/G Magnetic beads (Cat # 88802), Pierce Anti-Myc Magnetic Beads (Cat # 88842) were bought from Thermo Fisher Scientific (Waltham, MA). To stain cell plasma membranes for confocal microscopy images, CellBrite^™^ Fix 555 (Cat # 30088-T, 1:1000 for IF) and CF^®^ 405M (Phalloidin conjugate; Cat # 00034-T, 1:200 for IF) was obtained from Biotium (Fremont, CA) and mouse anti-ZO-1 from Thermo Fisher Scientific (Cat #33–0100). GLUT4 antibodies (Clone LM048, 10µg/mL for IF) used for detecting endogenous mouse GLUT4 by immunofluorescence was purchased from Integral Molecular (Philadelphia, PA) ^72^. The anti-cardiac troponin T antibody (ab8295) used for cardiomyocyte verification was purchased from Abcam (Cambridge, United Kingdom). The mouse-anti FLAG antibody (Clone M2, Cat # F1804–200UG, 1:1000 for WB; 1:200 for IF), anti-FLAG M2 magnetic beads (Clone M2, Cat # M8823) and VSV-G antibody (Cat# V4888, 1:1000, Sigma-Aldrich) was purchased from Sigma-Aldrich (St. Louis, MO). The goat anti-RIG-I antibody (Cat # sc-48931, 1:100 for IF) was a product of Santa Cruz Biotechnology (Dallas, TX) and rabbit anti-RIG-I antibody (Cat # 700366, 5µg/mL for IF) was from Invitrogen (Waltham, MA). The anti-*α tubulin* (Cat # 3873S, 1:2000), Actin (Cat # 4970L, 1:2000), RIG-I (Cat # 3743S, 1:1000), MDA5 (Cat # 5321S, 1:1000), phosphorylated IRF3 (Cat # 4947P, 1:1000) and total IRF3 (Cat # 11904P, 1:1000), rabbit-anti mouse MAVS IP antibody (Cat # 83000S, 1:50), UBXN9 (Cat # 2049S, 1:1000), phosphorylated TBK1 (Cat # 5483S, 1:1000), ISG15 (Cat # 2743, 1:1000), LDHA/C (Cat # 3558S, 1:1000), Caveolin (Cat # 3267T, 1:1000), phosphorylated AKT (Cat # 4060T, 1:1000), total AKT (Cat # 9272, 1:1000), phosphorylated c-Cbl (Cat # 8869T, 1:1000), total c-Cbl (Cat # 2747S, 1:1000), phosphorylated AS160 (Cat # 8881, 1:1000), total AS160 (Cat # 2670, 1:1000), total AMPK (Cat # 2793, 1:1000), and phosphorylated AMPK (Cat # 2535, 1:1000) were all purchased from Cell Signaling Technologies (Danvers, MA). Antibody used to detect UBXN9 cleavage (directed against C-terminus) was described previously ^17, 19^. Immunofluorescence secondary antibodies including Goat anti-rabbit Alexa Fluor^™^ 594 (Cat # A11037, 1:200), Goat anti-human Alexa Fluor^™^ 488 (Cat # A11013, 1:200), Donkey anti-goat Alexa Fluor^™^ 594 (Cat # A11058, 1:200), Donkey anti-goat Alexa Fluor^™^ 488 (Cat # A11055, 1:200), Donkey anti-rabbit Alexa Fluor^™^ 488 (Cat # A21206, 1:200) and DAPI solution (Cat # D1306, 1:1000) were purchased from Thermo Fisher Scientific (Waltham, MA). TransIT-X2 dynamic delivery system (Cat # MIR6005) was obtained from Mirus Bio, LLC (Madison, WI). IFN stimulatory DNA derived from Listeria monocytogenes genome (ISD, Cat# tlrl-isdn), 5’ triphosphate hairpin RNA (Cat# tlrl-hprna), and high molecule weight polyinosine-poly cytidylic acid [HMW poly (I:C),1.5–8 kb, Cat# tlrl-pic] and mouse IFN-β ELISA kits were from Invivogen (San Diego, CA). The recombinant mouse IFN-β was from R&D systems (Cat # 824-MB-010/CF). Glucose uptake (2-NBDG, Item # 600470) and lactate concentrations (Cat # J5021) was quantified using kits from Cayman Chemical (Ann Arbor, MI) and Promega (Madison, WI), respectively. All primers used in this study were obtained from Integrated DNA Technologies, Inc. (Coralville, IA).

### Isolation and culture of primary skeletal myoblasts and bone-marrow derived macrophages (BMDMs)

Primary skeletal myoblasts were isolated from *Ubxn9*^+/+^ and *Ubxn9*^−/−^ mouse quadricep muscle tissue, purified and differentiated as previously described with minor adjustments ^73^. Quadriceps (vastus medialis and vastus lateralis) were excised from two hindlegs of mice and placed in 1X phosphate-buffered saline (PBS) for washing. Muscle was then finely minced into small pieces, transferred to a 15ml conical tube, and spun down at 21,130 x g for 30s at RT to collect muscle pieces. Muscle was digested in Collagenase II (Cat #: 17101015, Gibco^™^) digestion media (400 U/ml in DMEM -FBS, sterile filtered) on a shaker at 100 rpm for 1.5 hours with vortexing halfway through digestion time. After shaking, vortex for 15s and muscle mix should appear cloudy. Tubes were then spun down at 1,400 x g for 5 min at RT, at which time supernatant was removed and muscle was resuspended in complete DMEM to neutralize collagenase II. To release myoblasts from muscle, tissue was pipetted up and down 30X using a sterile 10ml pipette and then strained over a 70µm strainer on a 50 ml tube. Cells were collected and strained through a 40µm filter to remove macrophages. Tubes were spun down at 1,400 x g for 5 min at RT and myoblast pellet resuspended in myoblast growth medium (MGM: DMEM/F12 + 1% p/s, 20% FBS, 10ng/ml basic fibroblast growth factor). Cells were seeded on plates precoated with 10% Matrigel (Cat #: 354234, Corning^™^).

For purification of a pure myoblast culture, digested muscle/cells were allowed to attach to Matrigel-coated plates for 72h without media removal. Three-days later, small, droplet-shaped myoblasts and larger fibroblasts should be visualized. Here, cells are ready for first pre-plating step. MGM medium was removed, and cells washed 2X with MGM to remove lingering cell debris and trypsinized to detach cells. Cells were centrifuged at 800 x g for 5 min and plated in 6-well plates that were not coated with Matrigel for 1h. This step is critical to remove contaminating fibroblasts as these will attach to the uncoated surface and myoblasts will remain in suspension. After 1h, fibroblasts were visualized on plate surfaces and supernatant containing myoblasts was removed and added to a new 10% Matrigel-coated plate. These purification steps were performed every 48h until > 98% myoblasts were obtained (typically ~ 3 pre-plate steps are necessary). After a pure culture is achieved, cells are ready for experimentation, or alternatively, they were differentiated into myotubes using differentiation medium (DM: DMEM + 2% horse serum) once cells reached ~ 80–90% confluency. DM medium was changed every 48h until myoblasts align and fusion begins (~ 120h). At this point, cells were ready for experimentation. Skeletal muscle myoblasts were isolated from *Slc2a4*^+/+^ and *Slc2a4*^−/−^ using the same protocol as above.

Bone marrow-derived macrophages were isolated and cultured from *Ubxn9*^+/+^ and *Ubxn9*^−/−^ mice based on our previous studies ^74^. In brief, bone marrow cells were differentiated into macrophages (BMDM) in L929-conditioned medium (RPMI 1640, 20% FBS, 30% L929 culture medium, 1x antibiotics/antimycotics) in a 10cm Petri dish at 37°C, 5% CO_2_ for 5–7 days. The culture medium was replaced fresh every 2 days. Attached BMDMs were dislodged by pipetting or gentle scrapping and counted for plating in plating medium (RPMI 1640, 10% FBS, 5% L929-conditioned medium, 1x antibiotics/antimycotics).

### Human iPSC culture and cardiomyocyte differentiation

Human iPSCs were generated as previously described ^75^. Briefly, the fibroblasts were derived from discarded female neonatal skin tissue under Yale Institutional Review Board approval. CytoTune^™^-iPSC 2.0 Sendai Reprogramming Kit (ThermoFisher Scientific, A16517) was used to reprogram fibroblast cells into iPSCs (Y6-iPSC). Twenty-four hours after viral transduction, infected cells were harvested and plated onto mitotically arrested MEF feeder layer with human iPSC medium (20% KSOR in DMEM/F12 medium supplemented with 10ng/ml bFGF), 1% non-essential amino acid (v/v), 2 mM L-Glutamine, 0.44 µM beta-Mercaptoethaol, and 1% Pen/Strep (v/v) (all from ThermoFisher Scientific, USA). The medium was changed every other day for three weeks. Colonies of iPSCs were then picked and expanded on MEF feeder layers for cardiac differentiation.

The iPSCs (Y6-iPSC) were grown to 70–80% confluency on MEFs and then dissociated using 1mg/mL Dispase II (Gibco,17105041) for 7–9 min. At which time, the iPSCs were further mechanically dissociated using a 5 mL pipette, collected, and centrifuged at 200 rpm for 4 min. The top supernatant was discarded to deplete the MEF cells, and the iPSC pellet resuspended in mTeSR^™^ 1 (Stemcell Technologies, 05850) containing 5µM ROCK inhibitor (Y-27632 dihydrochloride, Tocris Bioscience; 1244) and re-plated 1:1.5 on Matrigel-coated plates (Corning, diluted 1:60 in DMEM). Once reaching 80–95% confluency, cardiac differentiation was initiated using 20µM CHIR99021 (Selleckchem, S2924) in RPMI-1640 supplemented with 1% B27 minus insulin (Gibco, A18956–01) and one volume mTeSR^™^ 1 (considered day 1 of culture), as described in ^28^. On day three, medium was supplemented with 1% B27 minus insulin with the addition of 5µM IWP-4 (Stemgent Reprocell, 04–0036). After two days, the medium was switched to 1% B27 minus insulin without additional factors. Beating cardiomyocytes were typically observed on day 9–11. On day 11, medium was changed to 1% B27 containing insulin (Gibco, 17504044). Noncardiomyocytes were next eliminated starting from day 13 by treating cells with 4mM lactate (Sigma-Aldrich; L7022) in DMEM without glucose (Gibco; 11966025) for 4 days, with a medium change every other day. Enriched cardiomyocytes were then dissociated into clusters using a mixture of 10µg/mL Collagenase A and B (Roche, 10103586001 and 11088807001) for 30 min at 37°C. Cells were further dissociated into single cells using Accutase^™^ (Sigma-Aldrich; A6964) for 10 min at 37°C. Single cardiomyocytes were then plated onto fibronectin-coated plates. The RPMI1640 medium containing 1% B27 with insulin was replaced every other day for 2 weeks. On day 35, cardiomyocytes were processed for immunofluorescence (cardiac troponin T, cTnT) and siRNA/EMCV experiments.

### Mouse infection and disease monitoring

*Ubxn9* ^+/+^ and *Ubxn9*^−/−^ mice were intraperitoneally injected with 100 PFU of EMCV and morbidity and mortality were monitored twice a day for survival studies. Prior to infection, mice were fasted overnight to collect baseline IFN-β, glucose, lactate, and viral RNA levels (considered cutoff for the level of detection by qPCR). Mice were then allowed to feed *ad libitum* following infection. IFN-β in the plasma was analyzed at 24 h post-infection (hpi) from mice injected with 1000 PFU EMCV. Viral titers in plasma and heart tissue at 3 days post-infection (dpi) and 4dpi were assessed by qPCR and plaque assay, respectively. Lactate concentrations were quantified from the same samples used for qPCR of viral RNA and quantified using a Lactate-Glo Assay (Promega, Madison, WI, Cat # J5021).

### Cell culture and viruses

EMCV (Cat # VR129-B) and VSV (Indiana Strain, Cat # VR-1238) were purchased from American Type Culture Collection (ATCC) (Manassas, VA) and the multiplicity of infection was specified in each figure legend. ONNV UgMP30 strain (NR-51661) was provided by BEI Resources. Green fluorescence protein (GFP)-VSV was made by inserting a VSV-G/GFP fusion sequence between the VSV G and L genes and propagated in our lab for use in several studies ^25, 74, 76^. These viruses were propagated in Vero cells and titrated by a plaque forming assay.

HEK293T cells (Cat# CRL-3216), Vero cells (monkey kidney epithelial cells, Cat# CCL-81), C2C12 (mouse muscle cells, Cat # CRL-1772) and A549 (human lung cell line, Cat #CCL-185) were obtained from ATCC (Manassas, VA). The 3T3-L1 Myc-GLUT4-GFP fibroblast/adipocyte cell line was generated in our studies previously ^34, 77^. All cell lines were grown in Dulbecco’s modified Eagle medium (DMEM, Life Technologies, Grand Island, NY) supplemented with 10% FBS fetal bovine serum (FBS) and antibiotics/antimycotics. These cell lines are not listed in the database of commonly misidentified cell lines maintained by ICLAC and have not been authenticated in our hands. They are routinely treated with MycoZAP (Lonza) and tested for mycoplasma contamination in our hands. Cells were maintained in between experiments using stock DMEM supplied by Life Technologies (Grand Island, NY). During experiments with virus, ligands, etc., cells were cultured in base DMEM without glucose, sodium pyruvate or HEPES (Thermo Fisher Scientific, Cat # 11966025) supplemented with 10% FBS. Glucose was then complemented back at the same concentration (25mM) normally present in DMEM. This was to account for sodium pyruvate that feeds lactate and would skew glucose uptake/glycolysis measurements. For bioassay, 2fTGH-ISRE reporter cells were used to determine the concentration of IFN-β in the cell supernatant of A549 cells ^76^.

For differentiation of myotubes from C2C12 myoblasts, cells were seeded at ~ 70% confluency the day before beginning protocol. When cells reached ~ 95–100% confluency, culture medium was replaced with DMEM + 2% horse serum (HS) and medium replaced every 48h for 5–6 days, or until most of cells were fused together. Experiments were then carried out on myotubes. Murine 3T3-L1 fibroblasts were allowed to reach confluency at least two days prior to induction of differentiation into adipocytes. Differentiation was induced on the first day with DMEM containing 0.25 µM dexamethasone, 160 nM insulin, and 500 µM methylisobutylxanthine, followed by feeding cells with fresh medium containing 160nM insulin for two more days. On day four, cells were maintained in DMEM + 10% FBS until 8 days. Experiments were then performed on differentiated adipocytes ~ 8–10 days after process was initiated.

### Ligand treatments, siRNA, and in vitro virus infection

PRR ligands were transfected into cells with TransIT-X2 (Madison, WI, Product # MIR6005) using standard procedures described in product manual. In brief, DNA or RNA agonists were mixed with TransIT-X2 in 1X Opti-MEM (Gibco|Thermo Fisher Scientific, Waltham, MA, Ref # 31985–070) for 20 min before transfecting mixture dropwise onto cells. The concentrations of high-molecular-weight polyinosine-polycytidylic acid [HMW Poly(I:C)] (InvivoGen, San Diego, CA, Cat # tlrl-pic), 5’triphosphate (3p-hpRNA) (InvivoGen, San Diego, CA, Cat # tlrl-hprna-100) and interferon stimulatory DNA (ISD) (InvivoGen, San Diego, CA, Cat # tlrl-isdn) used for *in vitro* stimulation were specified for each experiment in the figure legend.

Knockdown of GLUT4 (*Slc2a4*) in C2C12 cells was done as described in user manual (Origene, Rockville, MD, Cat # SR416343). In brief, three unique targeting siRNA’s for *Slc2a4* (final concentration: 50nM) were mixed with TransIT-X2 in 1X Opti-MEM for 20 min before transfecting mixture dropwise onto cells. After 48h of incubation to achieve knockdown, cells were then treated with 3p-hpRNA as described above. A universal, non-specific scramble siRNA was used as a non-targeting control. The same protocol was adopted for knockdown of *Ubxn9* (Origene, Rockville, MD, Cat # SR417176). For iPSC-CMs, knockdown of human GLUT4 using two unique siRNA’s (final concentration: 50nM) were prepared based on the user manual (Origene, Rockville, MD, Cat # SR304402) and transfected as above.

For viral infection, viruses were diluted in DMEM without FBS and allowed to attach and infect cells for 2h; the cells were then washed with 1X phosphate-buffered saline (PBS) once and incubated with fresh medium. The MOI and infection time were denoted in each experimental figure legend.

### Plaque-forming assay

Quantification of infectious EMCV (Cat# VR-129B) viral particles in heart tissue/cell culture supernatant was performed on Vero cell monolayer with minor modifications ^74^. Briefly, heart tissue was weighed and 15mg of heart was digested in PBS using a Bio-Gen PRO200 Homogenizer (Pro Scientific, Oxford, CT, Cat # 01–01200). A total of 30–100 µg (total proteins) of tissue lysate or cell supernatant was serially diluted in DMEM (-) FBS and applied to confluent Vero cells (12-well plate) at 37°C for 2h. The inoculum was then removed and replaced with 2 ml of DMEM complete medium with 1% SeaPlaque agarose (Lonza, Cat# 50100). Plates were inverted at 37°C, 5% CO_2_ and plaques visualized using Neutral red (Sigma-Aldrich) after 24h of incubation. Viral titers were expressed as plaque forming units (PFU) / mL or gram of tissue.

### Plasmid construction and molecular cloning

The pB retrovirus vector containing GLUT4*myc*7-GFP (herein referred to as pGLUT4-GFP) was used in our studies previously ^77^. This plasmid was overexpressed in C2C12 using similar transfection methods described previously. The human full length (FL) and R169A point mutant GLUT4 were provided by Chuangye Yan at Tsinghua University ^41^. The FL (NCBI accession: NM_001042) and R169A variant were cloned into a new, custom pcDNA3.1-FLAG vector ^74^ for expression as an N-terminal FLAG-fusion protein using standard PCR amplification and cloning techniques. The pcDNA3.1-FLAG vector was also used to generate FLAG-UBXN9 ^76^. To generate GLUT4 truncations on the same plasmid backbone, the FL GLUT4 plasmid served as a template to clone GLUT4 ΔN24, ΔL6, ΔC42 mutants (PCR primers from Integrated DNA Technologies, Coralville, IA) and these were subsequently cloned using the same protocol. The pcDNA-FLAG-GLUT4 plasmids were transformed into *E. coli* DH5α (Thermo Fisher Scientific, Cat # 18265017) bacteria by electroporation at 42°C for 45sec. *E. coli* were plated on LB Agar containing 100µg/mL ampicillin and inverted overnight at 37°C. Antibiotic-resistant colonies were picked and grown in LB media at 37°C overnight by shaking (100rpm). Plasmid DNA was extracted using PureLink^™^ Quick Plasmid Miniprep Kit (Thermo Fisher Scientific, Cat # K210011). Sanger sequencing was used to verify GLUT4 insertion into plasmids. RIG-I mutant plasmids were similarly cloned as above where the human FL RIG-I inserted into the pcDNA3.1-FLAG vector ^74^ served as the template to clone RIG-I CARD, Helicase, ΔCARD and CTD. MDA5 FL was also cloned into the pcDNA3.1-FLAG vector and served as the template to clone MDA5 CARD and ΔCARD plasmids.

### Generation of gene knockout cell lines with CRISPR-Cas9 technology

Pre-designed, gene specific guide (g) RNAs (Integrated DNA Technologies, Coralville, IA) were subcloned into a lentiCRISPR-v2 vector ^78^ and correct insertion was confirmed by sequencing. To generate lentiviral particles, each gRNA vector was transfected into HEK293T cells with the packaging plasmids pCMV-VSV-G and psPAX2 (Didier Trono lab via Addgene, Watertown, MA, Cat # 12259). After 24h, half of the cell culture medium was replaced with DMEM, and viral particles were collected at 48–72h after transfection. The viral supernatant was cleared by brief centrifugation at 2000 rpm for 5min. C2C12 target cells were then seeded at ~ 50% and transduced the next day with each individual lentivirus. The WT control was lentiCRISPRv2 vector only. C2C12 cells were then selected with 0.8 µg/mL of puromycin for 10–20 days, changing puromycin media every other day. Successful knockout clones were confirmed by western blotting. Guides targeting mouse UBXN9 (F-CACCGATGAAGTGCTACGACCCCGT; R-5’ AAACACGGGGTCGTAGCACTTCATC) GLUT4 (F-5’ CACCGAGGCACCCTCACTACGCTCT; R-5’ AAACAGAGCGTAGTGAGGGTGCCTC), LDHA (F-5’ CACCGTGTTCACGTTTCGCTGGACC; R-5’ AAACGGTCCAGCGAAACGTGAACAC), and AKT2 (F-5’ CACCGTCACAAAGCATAGGCGGTCA; R-5’ AAACTGACCGCCTATGCTTTGTGAC) were used in this study.

### Purification of total cellular RNA, reverse transcription and quantitative (q) PCR

Approximately 15mg of mouse tissue, ~ 20 µL of whole blood and up to 1x 10^6^ culture cells were collected in 300 µL of lysis buffer (RNApure Tissue & Cell Kit, CoWin Biosciences, Cambridge MA, United States). Heart tissue was homogenized as described, mixed with lysis buffer, and extracted according to the product manual. Isolated RNA was quantified using a spectrometer feature on BioTek Cytation 1 imaging reader (Agilent, Santa Clara, CA) and RNA concentration was normalized according to the lowest concentration across all samples with RNase-free water. RNA samples were normalized and converted into cDNA using the PrimeScript^™^ Reverse Transcription (RT) reagent Kit (TaKaRa Bio, Inc, Cat# RR037A). Quantitative PCR (qPCR) was performed with gene-specific primers and iTaq Universal SYBR Green Supermix (BioRad, Cat# 1725124). Results were calculated using the 2^–ΔΔCt^ method from the C^T^ values for each sample and gene of interest. A housekeeping gene (*Actb*, *Polr2b*) was used as an internal control. The qPCR primers have been used and validated in our previous studies ^74, 79^.

### Mouse glucose tolerance test (GTT)

*Ubxn9* ^+/+^ and *Ubxn9*^−/−^ mice were fasted overnight (~ 12h) followed by being administered with 1 mg glucose/kg body weight (Sigma, St. Louis, MO, Lot # 82H0725) via intraperitoneal (i.p.) injection. Tail vein blood at baseline and indicated time points after injection was collected and measured for glucose levels by using a handheld glucometer (Germaine Laboratories, San Antonio, TX). Glycemia was reported as mg/dl.

### 2-deoxyglucose (2-DG) and lactate assays

Cells were plated in 96-well clear bottom black plates one day before assay to allow equilibration in metabolism. To quantify 2-NBDG uptake following insulin treatment, protocol was followed according to manufacturer’s instructions, with minor modifications (Cayman Chemical, Ann Arbor, MI, Item # 600470). For acute glucose uptake, cells were serum starved in no glucose (-FBS) DMEM medium for 3h before 50 µg/ml 2-NBDG was added to no glucose DMEM containing FBS and 200nM human insulin (Millipore Sigma, Darmstadt, Germany, Cat # 91077C) for 15–20 min. Supernatant was removed and cells were washed 2X with assay buffer to remove non-specific binding. After second wash, cells were placed in assay buffer and intracellular 2-NBDG taken up by cells was detected with fluorescent filters (485nm/535nm) on BioTek Cytation 1 imaging reader (Agilent, Santa Clara, CA). To account for background signal, no insulin treated cells were used as “steady state” glucose consumption and 2-NBDG in DMEM was added to empty wells without cells. Both controls were subtracted from insulin treated groups to calculate relative glucose uptake. For experiments of longer duration (3p-hpRNA, virus, IFN-β), 2-NBDG was added to DMEM without glucose with or without specified treatment and incubated with cells until timepoint indicated in figure legends. To account for cell growth/background, “Mock” cells were incubated with 2-NBDG in DMEM alone and assayed in tandem with treated cells. Cells were washed and assayed as described above for insulin treatment. For testing inhibition of glucose uptake, cells were pretreated for 1h with 50µM of Fasentin (R&D Systems, Minneapolis, MN, Cat # 6100/10) diluted in DMEM without glucose (or DMSO control). Media was then replaced containing fresh inhibitor with 100 µg/ml 2-NBDG and cells were incubated for 1h before assessing glucose uptake as described previously.

Lactate was detected using a Lactate-Glo Assay (Promega, Madison, WI, Cat # J5021)

for extracellular supernatant (cell culture medium), intracellular (cell lysates) and mouse plasma. Protocol was followed exactly as suggested in product manual as each type of sample required unique processing. For intracellular lactate, cells were plated in a white 96-well clear bottom plates and treated as described in figure legends. After specified timepoints, supernatant was removed, and lysate used for determining intracellular lactate concentration. For mock groups, cells were incubated in media side-by-side with experimental wells and processed simultaneously. Mouse plasma was diluted 100-fold and processed as for cell culture supernatant samples. Plates were measured using a BioTek Cytation 1 imaging reader (Agilent, Santa Clara, CA) with luminescent filters. DMEM + 10% FBS was used as background control for cell culture supernatant and plasma; 1X PBS used for intracellular lactate background.

### Enzyme-linked immunosorbent assay (ELISA) and immunoblotting

IFN-β in cell supernatants, homogenized tissue and plasma were assessed using LumiKine^™^ Xpress mIFN-β 2.0 ELISA detection kit according to manufacturer’s instructions (InvivoGen, San Diego, CA, Cat #luex-mifnbv2). Human IFN-β in cell supernatants of iPSCs were assessed using DuoSet^®^ ELISA kit according to the manufacturer’s instructions (R&D Systems, Minneapolis, MN, Cat #DY814–05). The IFN-β data are presented as pg/ml. For western blotting analysis, cells were lysed in RIPA (Alfa Aesar, Tewksbury, MA, Cat # J63306) or for overexpression/detection of GLUT4, 2% n-Dodecyl-β-D-Maltopyranoside (Anatrace, Maumee, OH, Cat # D3101GM). For detection of GLUT4, samples were not boiled and lysed on a rotator for 2h at 4°C before centrifuging at 21,000 x g for 10min at 4°C. Samples were run on standard dodecyl sulfate-polyacrylamide gels and transferred onto nitrocellulose membranes using Mini Trans-Blot^®^ Cell transfer systems (Biorad, Hercules, CA, Cat #1703930). Blots were then blocked with 5% milk in TBS-T and probed with appropriate primary and secondary antibodies. Protein bands were visualized with enhanced chemiluminescent (ECL) substrate (Lumigen, Southfield, MI, Cat # TMA-100) on a ChemiDoc^™^ MP Imaging system (Biorad, Hercules, CA, Cat # 12003154).

### Co-immunoprecipitation

HEK293T cells were transfected with GLUT4 expression plasmids or RIG-I/MDA5 mutant plasmids using the TransIT-X2 system as described above. Total cell lysates were prepared from transfected cells in lysis buffer containing [ 2% n-Dodecyl-β-D-Maltopyranoside (DDM), 25mM HEPES, 150mM NaCl and 1X protease inhibitors] by gentle scrapping and pipetting before transferring to a 1.5ml centrifuge tube. Lysate was rotated at 4°C for 2h and then centrifuged at 21,000 x g for 10min. Supernatant was removed and again rotated overnight with 20µl of pre-washed (25mM HEPES, 150mM NaCl) anti-FLAG magnetic beads at 4°C. Co-immunoprecipitation was then preformed according to manufacturer’s instructions (Sigma Aldrich, St. Louis, MO, Cat # M8823). IP elution was mixed with 4X SDS loading buffer. Samples were not boiled to maintain GLUT4 transmembrane conformation. For harder to transfect cells such as C2C12, GLUT4 overexpression was carried out in cell suspension. Briefly, cells were dislodged by trypsin digestion and pelleted by brief centrifugation at 800 x g for 5min. The cell pellet was then resuspended in transfection mix (DNA + TransIT-X2 + 1X Opti MEM) prepared as described above for 7–10 min with intermittent agitation at 37°C. Prewarmed DMEM was then added and plated for further culture. FLAG immunoprecipitation then proceeded as above.

Myc immunoprecipitation was performed using 3T3-L1 adipocytes stably expressing the Myc-GLUT4-GFP reporter. Total cell lysates were prepared in lysis buffer (50mM Tris HCl, 150mM NaCl, 1mM EDTA and 1% TRITON X-100) by gentle scrapping and pipetting before transferring to a 1.5ml centrifuge tube. To maintain GLUT4 conformation, lysate was kept on ice for 30min with intermittent vortexing every 5min. Lysate was then centrifuged at 10,000 x g for 10min. Supernatant was removed and mixed with 20µl of pre-washed (25mM Tris, 150mM NaCl and 0.05% Tween-20) Pierce Anti-Myc Magnetic Beads and rotated overnight at 4°C. Co-immunoprecipitation was then preformed according to manufacturer’s instructions (Thermo Fisher Scientific, Waltham, MA, Cat # 88842). IP elution was mixed with 4X SDS loading buffer. Samples were not boiled to maintain GLUT4 transmembrane conformation.

### Endogenous MAVS co-immunoprecipitation

For detecting endogenous RIG-I: MAVS interactions, ~ 6 x 10^6^ cells were lysed in 500µl of IP lysis buffer (50mM Tris HCl, 150mM NaCl, 1mM EDTA and 1% TRITON X-100). Cell debris was clarified by centrifuging at 7000 x g for 15min at 4°C and supernatant then mixed with rabbit anti-MAVS (Cell Signaling Technology, Danvers, MA, Cat # 24930) diluted 1:60 (~ 550ng antibody). Lysate and antibody were rotated overnight at 4°C. The antigen sample/antibody mixture was then added to 1.5ml microcentrifuge tube containing 20µl (0.20mg) of prewashed Pierce Protein A/G Magnetic beads (Thermo Fisher Scientific, Waltham, MA, Cat # 88802) and incubated at RT for 2h with mixing. Beads were collected with a magnetic stand and washed thoroughly 3X (1x TBS) as detailed in user manual. Target complexes were eluted in 100µl SDS-PAGE sample lysis buffer on a rotator for 20min. Beads were collected with a magnetic stand and remaining elution was boiled for 10min. To promote RIG-I: MAVS interactions, 3p-hpRNA was transfected into cells as described in “Ligand treatments” section for indicated times detailed in figure legends; reconstitution water diluted to the same ratio in transfection mixture was used for mock groups.

### Endogenous MAVS aggregation

To detect MAVS oligomers, C2C12 cells were seeded in 6-well plates at a density of ~ 1 x 10^6^ per well in duplicate and treated as denoted in figure legends. After treatment, cells were washed with 500 µL cold PBS before being lysed in 2% DDM lysis buffer (n-Dodecyl-β-D-Maltopyranoside, 25mM HEPES, 150mM NaCl and 1X protease inhibitors] by gentle scrapping and pipetting. Lysate was then rotated at 4°C for 2h and centrifuged at 21,000 x g for 10min to remove cell debris. Next, supernatants were transferred and mixed with 20 µL Native Sample Buffer (Bio-Rad #1610738) that was then loaded into a 4–16% NativePAGE^™^ (Invitrogen^™^ #BN1004BOX). The samples were then run in an Invitrogen NuPage^®^ Novex^®^ Gel System at 120 V for 1 h, 180 V for 30 min, 240 V for 30 min and then 300 V for 1 h in sequence (to maintain at 9 mA electricity). Gels were then transferred to nitrocellulose membranes using Mini Trans-Blot^®^ Cell transfer systems (Biorad, Hercules, CA, Cat #1703930). Membranes were then blocked with 5% milk in TBS-T, probed with appropriate primary overnight and proteins detected with HRP-conjugated secondary antibodies. Protein bands were visualized with enhanced chemiluminescent (ECL) substrate (Lumigen, Southfield, MI, Cat # TMA-100) on a ChemiDoc^™^ MP Imaging system (Biorad, Hercules, CA, Cat # 12003154).

### Plasma membrane fractionation

The plasma membrane fraction was isolated from GLUT4^+^ C2C12 myocytes and 3T3-L1 adipocytes as previously described, with modifications according to experimental goals ^77, 80^. Cells were grown on 6-well plates to ~ 2–3 x 10^6^ and serum starved for 3h before 20min of insulin treatment or transfected with 3p-hpRNA for 6h to stimulate innate immune receptors. Culture medium was removed, cells washed with cold 1X PBS twice and then lysed in 180µl Buffer A (50mM Tris HCl, 0.5mM DTT, 0.1% NP-40, 1X protease inhibitors). Cells were scrapped off plates and transferred to a 1.5ml centrifuge tube on wet ice before homogenized with a handheld electric pestle for 15sec (Kimble Chase LLC, United States). The homogenized lysate was passed 3X through a 23G needle attached to a 1mL syringe to shear DNA/nucleus and liberate intracellular proteins (small aliquot removed for total cell lysate and subsequently lysed in RIPA). Current and subsequent centrifugation steps were all done at 4°C to maintain plasma membrane protein structures and prevent denaturing. The remainder of the sheared lysate was centrifuged at 200 x g for 1 min and supernatant transferred to a new pre-chilled tube (Supernatant 1); cell pellet was again homogenized in 90µl Buffer A (0.1% NP-40) until fully dispersed followed by centrifugation for 1 min at 200 x g. Supernatant 1 was combined with Supernatant 2 and the pellet material was discarded, which contained only cell debris. The plasma membrane proteins in supernatant 1 + 2 were centrifuged for 10min at 750 x g to isolate the post-plasma membrane (PPM) fraction and supernatant was then stored on ice for later processing. Again, the cell pellet was suspended in 90µl Buffer A (0.1% NP-40) by vigorously vortexing for 10 sec and centrifuged for 10 min at 750 x g. Cell pellet was resuspended with 45µl Buffer A containing 1% NP-40 (50mM Tris HCl, 0.5mM DTT, 1% NP-40) and incubated on ice for 1h with occasional mixing to solubilize membrane proteins. After 1h, proteins were centrifuged for 20min at 12,000 x g and supernatant containing pure plasma membrane (PM) proteins was removed and mixed with SDS-PAGE sample lysis buffer. The backup PPM fraction was incubated on ice for 1h with occasional mixing and centrifuged at 12,000 x g and mixed with SDS-PAGE sample lysis buffer. Total cell lysate (TCL) was incubated with RIPA for 60 min and centrifuged at the same conditions with PM and PPM fractions. Caveolin was used as loading control for the PM fraction and GAPDH for TCL.

### Subcellular fractionation

Subcellular fractionation by differential centrifugation was performed as previously described ^38^, with slight modifications. Briefly, myotubes were washed 1X with PBS before adding sucrose homogenization buffer (250mM sucrose, 50mM Tris-HCl, 5mM MgCl_2_ and 1X protease inhibitor cocktail) and scrapping cells off plates. Cellular fractions were kept on ice or 4°C for the remaining steps. Cells were broken up with a Dounce homogenizer for 5min (or until cells were observed to be > 90% broken) and then the nuclei/unbroken cells were vortexed for 15sec and centrifuged at 800 x g for 15min. The pellet discarded and the supernatant was again centrifuged at 800 x g for 10min. The supernatant was retained and centrifuged at 11,000 x g for 10min to separate the cytosol fraction (supernatant) and crude mitochondria (pellet). Crude mitochondria pellets were resuspended in 200µl sucrose buffer, centrifuged again at 11,000 x g for 10min, and pellets resuspended in lysis buffer (50mM Tris-HCl, 1mM EDTA, 0.5% Triton-X100 and 1X protease inhibitor cocktail). Mitochondrial lysis was sonicated on ice 3X in 5 sec increments with 30sec pauses. The lysis buffer was labeled “mitochondrial fraction’ and ready for SDS-PAGE. The cytosol fraction (containing cytosol and microsomes) were centrifuged at 100,000 x g for 1hr in an ultra-centrifuge. The cytosol in the supernatant was concentrated with 100% ice-cold acetone in −20°C for 1hr while the microsome pellet was discarded. The cytosolic fraction was again centrifuged at 12,000 x for 5min, and the pellet containing precipitated cytosolic proteins was resuspended in lysis buffer containing SDS, 10% β-mercaptoethanol and 8M urea.

### Immunofluorescence microscopy

Cells were cultured as experiment requested and treated as described in figure legends. For most experiments, C2C12 or 3T3-L1 adipocytes were differentiated in 8-well chambered microscopy slides precoated with IbidiTreat (Ibidi, Gräfelfing, Germany, Cat # 80826) to facilitate attachment for extended periods of time. For plasma membrane staining, medium was removed, and cells washed 2X with cold 1X PBS for 5 min per wash. Cells were subsequently stained for 15 min at 37°C with CellBrite^™^ Fix 555 (Cat # 300088-T) diluted 1:1000 in PBS. Experiments that did not require plasma membrane staining were immediately washed and fixed as described below. Cells were washed 1X with PBS for 5 min and fixed for 15 min with 4% paraformaldehyde in PBS at RT. Cells were then washed twice with cold 1X PBS for 5 min per wash and permeabilized with 0.1% TRITON X-100 for 15 min at RT. Permeabilization solution was removed and cells were washed 1X with PBS and blocked for 1h at RT in blocking buffer (1% BSA, 10% goat serum, 0.3M glycine, 0.25% TRITON X-100). Primary antibodies (anti-RIG-I, 1:100; anti-GFP, 1:100; anti-FLAG, 1:200) were diluted in antibody buffer (1% BSA, 0.3M glycine, 0.25% TRITON-X100) and incubated with cells overnight at 4°C in the dark. Cells were washed 3X with cold 1X PBS for 5 min per wash. A mix containing a variety of Alexa Flour^™^ -conjugated secondary antibodies diluted 1:200 (see “Reagent and antibodies” section for specific species reactivity and fluorophore) in antibody buffer was subsequently added to cells for 1h in the dark at RT. Cells were then washed 2X with cold 1X PBS and incubated with DAPI counterstain (Thermo Fisher Scientific, Cat # D1306) diluted 1:1000 in antibody buffer for 10 min in the dark at RT. DAPI was removed and cells were washed once for 5 min in 1X PBS. For chambered slides, cells were covered with 200µL of new 1X PBS; cover slips were mounted onto microscope slides if cells were processed on glass slides. Slides were imaged using a Zeiss 880 laser scanning microscope with a x63.0 oil objective lens.

Experiments involving detection of endogenous GLUT4 were processed as described in the literature and in consultation with Integral Molecular (Philadelphia, PA) ^33, 72^. C2C12 cells were differentiated in Ibidi chambered microscopy slides and treated as detailed in figure legends. Cells were washed three times with cold 1X PBS for 5 min per wash on ice. Cells were subsequently blocked with 10% goat serum in PBS for 30 min in ice before the addition of exofacial GLUT4 antibody at 10µg/mL in 10% goat serum (Clone LM048, Integral Molecular). Primary antibodies were incubated for 2h at 4°C in the dark and then washed thrice with cold 1X PBS. Cells were subsequently fixed with 4% paraformaldehyde in PBS for 5 min on ice and then moved to RT for an additional 20 min. After fixation, cells were washed twice with 1X PBS and incubated with 50mM glycine in PBS for 5 min at RT. Cells were washed twice with cold 1X PBS before the addition of mixture containing secondary antibody diluted 1:200 (g*oat* anti-human Alexa Fluor^™^ 488 Cat # A11013) and Hoechst 33342 (Thermo Fisher Scientific, Cat # 62249) diluted 1:10,000 in 10% goat serum for 1h at RT. Secondary antibody was removed and cells were washed three times with 1X PBS. For co-staining of extracellular GLUT4 and intracellular RIG-I, cells were first processed and stained with primary anti-GLUT4 as described above. Then, cells were fixed as before and permeabilized with 10% goat serum containing 0.2% saponin for 15 min at RT. Cells were subsequently blocked intracellularly with 10% goat serum for 30 min on ice and then incubated with primary antibody diluted to 5µg/mL *in* 1% BSA, 0.3M glycine and 0.1% saponin. Intracellular staining was allowed to proceed overnight at 4°C in the dark. After overnight incubation, cells were washed thrice with 1X PBS on ice and secondary antibody staining proceeded as before. At the secondary antibody step, phalloidin was used to mark the plasma membrane.

All microscopic images were first processed in the Zen 2.3 microscopy software (Zeiss Group, Jena, Germany) before further analysis in ImageJ (NIH). The same microscope instrument settings were used for all samples, and all images were analyzed using the same settings. A vector (white arrow) was drawn from the edge of the nucleus (DAPI) to the plasma membrane (CellBrite 555); the midpoint of the indexed arrow was then considered the perinuclear and peri-plasma membrane regions, respectively. The relocalization of RIG-I intensity (AF488) was calculated by the ratio of fluorescence in the perinuclear to the peri-plasma membrane regions. Fluorescence intensities of all three markers were then traced along the vector and distance (in µm) of RIG-I to the plasma membrane was calculated. Colocalization of membrane and RIG-I fluorescence was measured on a per-cell basis and Pearson’s coefficient calculated using the JACoP Plugin in ImageJ. Mean fluorescence intensities (MFI) for surface GLUT4 were calculated on a per-image basis and identical settings used for each image. In brief, images were adjusted using “Threshold” setting to fix a cutoff value as to highlight regions with fluorescence above the background intensity. Final pmGLUT4 MFI = MFI of cell-background MFI. Because GLUT4 antibody recognizes only exofacial GLUT4 and the cells were not permeabilized or fixed before staining, detected fluorescence was considered on the plasma membrane.

For immunostaining of iPSC-derived cardiomyocytes (iPSC-CMs), cells were fixed with 4% paraformaldehyde in PBS for 12 min at RT, washed with PBS and permeabilized with 0.01% Triton X-100 for 20 min at RT. iPSC-CMs were then blocked with 10% goat serum in PBS for 30 min at RT and stained for cardiac troponin T (cTnT) and nuclei with DAPI (SouthernBiotech, Cat #0100 − 20). Confocal fluorescent microscope (Leica; Multiphoton Microscope TCS SP8 MP) was used to image immunostained cardiomyocytes.

### RNA sequencing analysis from patient datasets

RNA sequencing datasets were obtained from PRJNA491748 (CIM study) and GSE143323 (DM study). For transcriptomic analysis of GSE143323, differentially expressed genes (DEGs) were re-identified using DEseq2. FDR/Benjamini-Hochberg method was used to adjust p-values and DEGs with the cutoff of FDR < 0.05 and Log_2_FC > 0.5 and used to generate volcano plots. Pathway analysis using QIAGEN Ingenuity Pathway Analysis (IPA) included 2361 down-regulated genes and 4417 up-regulated genes and all significant (p < 0.05) pathways with Z-scores were plotted. Selected pathways that were either activated (red) or suppressed (blue) were labeled. Pearson r correlations were conducted on FPKMs values.

### Graphing and statistics

All statistical analysis was performed using Graphpad Prism9 software. All experiments were performed in either duplicate or triplicate. Survival curves were analyzed using a log-rank (Mantel–Cox) test. For animal studies, an unpaired, two-tailed non-parametric Mann-Whitney *U* test was applied to statistical analysis. For comparison in which multiple variables were tested with multiple time points, two-way ANOVA analysis was performed. For comparison of two data points *in vitro* or *in vivo*, the student’s two-tailed unpaired Student’s *t* test was applied. The sample sizes (biological replicates), specific statistical tests used, and the main effects of our statistical analyses for each experiment were detailed in each figure legend. Asterisk coding is as follows: * p ≤ 0.05; ** p ≤ 0.01; *** p ≤ 0.001; **** p ≤ 0.0001. Data with error bars depict the average with the SEM.

## Supplementary Material

Supplement 1

## Data Availability

The datasets generated during and/or analyzed during the current study are available from the corresponding author upon request.
