# Supplementary material for: UBXN9 governs GLUT4-mediated spatial confinement of RIG-I-like receptors and signaling
Source: Res Sq. 2024 Jun 4:rs.3.rs-3373803. Preprint. [Version 1] doi: 10.21203/rs.3.rs-3373803/v1 (PMC11177981; doi:10.21203/rs.3.rs-3373803/v1)
Supplement: Supplement 1 [file NIHPPrs3373803v1-supplement-1.pdf]

Supplementary Files

This is a list of supplementary files associated with this preprint. Click to download.

- UBXN9GLUT4SupplementalFiguresV13FINALRESUBMISSION.pdf
